# Prognostic modeling of early-onset nondistal gastric cancer identifies *ARSB–PDCD1* ratio as an immune-related survival stratifier

**DOI:** 10.3389/fimmu.2025.1655106

**Published:** 2025-09-29

**Authors:** Zhiqiang Zhang, Xin Zhong, Qunlong Jin, Zhumei Chen, Yanming Yang, Youheng Jiang, Huaixiang Zhou, Caiyan An, Junjing Zhang, Yulong He, Zhang Fu, Kaiming Wu, Ningning Li

**Affiliations:** ^1^ Tomas Lindahl Nobel Laureate Laboratory, The Seventh Affiliated Hospital, Sun Yat-sen University, Shenzhen, China; ^2^ Digestive Diseases Center, Guangdong Provincial Key Laboratory of Digestive Cancer Research, The Seventh Affiliated Hospital, Sun Yat-sen University, Shenzhen, China; ^3^ Department of Anesthesiology, The Seventh Affiliated Hospital, Sun Yat-Sen University, Shenzhen, China; ^4^ Inner Mongolia Key Laboratory of Allergic Diseases, Foundational and Translational Medical Research Center, Hohhot First Hospital, Hohhot, China; ^5^ Department of Hepato-Biliary Surgery, Department of Surgery, Hohhot First Hospital, Hohhot, China; ^6^ Department of Geriatrics, The Seventh Affiliated Hospital, Sun Yat-Sen University, Shenzhen, China

**Keywords:** early-onset gastric cancer, nondistal gastric cancer, ARSB, PDCD1, prognostic model

## Abstract

**Background:**

Despite a global decline in gastric cancer (GC) incidence, nondistal GC (NDGC) is increasingly prevalent among younger patients, necessitating targeted investigation of early-onset NDGC (EONDGC) to identify prognostic determinants for enhanced risk stratification.

**Methods:**

EONDGC patients were identified from multiple datasets, including the Surveillance, Epidemiology, and End Results (SEER) database, the Cancer Genome Atlas (TCGA) Stomach Adenocarcinoma cohort, and the Affiliated Hospitals of Sun Yat-sen University (SYSU) as an external validation cohort. Propensity score matching was performed to reduce baseline differences between groups. A prognostic model was developed using univariate and multivariate Cox regression and LASSO analysis in a 7:3 training–validation split. The prognostic model was applied to TCGA patients to generate risk scores, and high-risk patients were selected for differentially expressed genes (DEGs) analysis. The identified genes were then analyzed using Cox regression and Kaplan-Meier methods to determine prognostic relevance. In parallel, MGC-803 and AGS cells were transiently transfected to overexpress *ARSB*; RT-qPCR verification, scratch and transwell migration assays quantified motility.

**Results:**

A total of 535 EONDGC patients from SEER and 171 from SYSU were included. The prognostic model, incorporating seven clinical variables (race, pathological grade, T, N, and M stage, lymph node ratio, and chemotherapy), achieved robust performance with concordance index values of 0.758 (training), 0.718 (validation), and 0.762 (SYSU), with all AUCs > 0.75. In the TCGA patients, 73 upregulated genes were identified from high-risk patients through DEGs analysis. Among these, *ARSB* and *PDCD1* were determined to be independent prognostic markers based on Cox and Kaplan-Meier analyses. Furthermore, a higher *ARSB/PDCD1* ratio (APR) was associated with poorer overall survival (*P* = 0.041). *In vitro*, *ARSB* overexpression increased scratch migration area and transwell-migrated cell counts versus empty vector.

**Conclusion:**

This study developed a clinical prognostic model for EONDGC and therefore identified *ARSB* and *PDCD1* as key molecular markers. The APR value enhances survival stratification, offering valuable insights into personalized prognosis and potential immunotherapy strategies.

## 1 Introduction

Gastric cancer (GC) remains a significant global health challenge. According to the World Health Organization in 2022, GC ranks fifth worldwide in both incidence and cancer-related mortality, accounting for 4.9% of all new cancer cases and 6.8% of cancer deaths (1).

Anatomically, GC is classified into distal GC (DGC), affecting the antrum and pylorus, or nondistal GC (NDGC), involving the cardia, fundus, and body of the stomach (2, 3). Different anatomical locations of GC influence its clinical presentation. DGC frequently presents with noticeable symptoms such as postprandial fullness, nausea, and vomiting caused by pyloric obstruction, making it more likely to be detected early (4, 5). In contrast, NDGC is often asymptomatic in its early stages or presents with mild symptoms like upper abdominal discomfort or dyspepsia, which are easily overlooked, complicating timely diagnosis.

Etiologically, DGC often arises from chronic multifocal atrophic gastritis of the antrum (6) and is strongly associated with Helicobacter pylori infection (7). In contrast, NDGC has a more complex etiology, including gastroesophageal reflux disease, obesity, and smoking (8–11). NDGC’s complex etiology poses challenges for prevention and management. Although the mortality rates of DGC and NDGC are comparable, recent epidemiological data indicate a rising incidence of NDGC, particularly in developed countries and among younger populations (12).

Notably, within NDGC, the incidence of early-onset gastric cancer (EOGC), defined as GC diagnosed before the age of 50 years (13, 14), has been increasing in recent years (15–17). This trend underscores the urgency of studying early-onset nondistal gastric cancer (EONDGC), a specific subtype of NDGC. In this study, our objective was to elucidate factors influencing survival outcomes in EONDGC. To achieve this, we performed a refined prognostic assessment of patients with EONDGC, identifying key clinical and molecular determinants that may better guide clinical decision-making and inform the development of individualized treatment strategies.

## 2 Methods

### 2.1 Data sources

This study utilized comprehensive datasets from three sources. Firstly, data from the Surveillance, Epidemiology, and End Results (SEER) Program (https://seer.cancer.gov/) provided population-based information on cancer incidence, mortality, and clinical outcomes from 17 U.S. registries spanning 2000 to 2019, extracted via SEER*Stat software (version 8.4.4). Secondly, HTSeq gene expression data from The Cancer Genome Atlas Stomach Adenocarcinoma (TCGA-STAD) cohort (https://www.cancer.gov/tcga), including 412 tumor samples and 36 normal controls, were downloaded via the TCGAbiolinks package for molecular analyses. Lastly, clinical data from 171 patients diagnosed and treated at the Affiliated Hospitals of Sun Yat-sen University (SYSU) between 2000 and 2024 served as an external validation cohort. Ethical approval for SEER and SYSU data usage was obtained as required; full ethical details are provided in the Ethics Statement section.

### 2.2 Inclusion criteria

Eligible patients met the following six conditions: (1) aged between 19 and 85 years; (2) confirmed diagnosis of GC; (3) survival months ≥ 1 month; (4) primary tumors located in the cardia, fundus, body, antrum, and pylorus (ICD-O-3 codes 16.0–16.4); (5) histological subtype of adenocarcinoma or signet ring cell carcinoma; and (6) disease-specific death attributed to GC.

### 2.3 Exclusion criteria

Patients were excluded on the basis of the following seven conditions: (1) age ≤ 18 years or > 86 years; (2) diagnosed with gastrointestinal stromal tumors, late-onset distal gastric cancer or neuroendocrine tumors; (3) survival months < 1 month; (4) incomplete pathological information, including TNM stage, tumor differentiation, tumor size, or number of positive lymph nodes; (5) missing demographic data such as marital status, race, or household income; (6) nongastrectomy or unknown treatment modalities; and (7) incomplete follow-up information.

### 2.4 Statistical analysis

All the statistical analyses and visualizations were conducted via R software (18) (version 4.4.1; R Foundation for Statistical Computing, Vienna, Austria). The following R packages were used: survminer, MatchIt, survival, rms, compareGroups, foreign, dcurves, survivalROC, pROC, DynNom, tidyverse, DESeq2 (19), pheatmap, and clusterProfiler. All tests were two-tailed, and a *P* value < 0.05 was considered to indicate statistical significance.

Propensity score matching (PSM) was performed via a logistic regression model (20). A 1:1 nearest-neighbor matching method was applied, and matching quality was evaluated via standardized differences. Three patient groups were derived: EONDGC, early-onset DGC (EODGC), and late-onset NDGC (LONDGC). Baseline characteristics were compared before and after PSM. Subgroup analyses were performed within the EONDGC group.

Continuous variables are presented as means ± standard deviations or medians with interquartile ranges, depending on distribution. Categorical variables are summarized as counts and percentages. Group comparisons were performed using the t-test, Wilcoxon rank-sum test, or chi-square test as appropriate (21).

### 2.5 Prognostic modeling

#### 2.5.1 Model construction and validation

EONDGC patients were randomly assigned to SEER-training and SEER-validation sets in a 7:3 ratio. In the training set, variables with *P* < 0.1 in univariate Cox regression were included in multivariate analysis, and the model with the lowest Akaike Information Criterion (AIC) was selected (22). The final prognostic model was constructed by integrating the multivariate Cox regression results with least absolute shrinkage and selection operator (LASSO) regression (23).

Discrimination was evaluated using Harrell’s concordance index (C-index), and time-dependent predictive performance was evaluated by the receiver operating characteristic (ROC) curves and corresponding the area under the curves (AUC) (24). Calibration was assessed via calibration curves. Clinical utility was evaluated using decision curve analysis (DCA) (25).

#### 2.5.2 Visualization and stratification

A prognostic nomogram was constructed in the training set, assigning scores to each prognostic variable based on its relative contribution to survival outcomes. The total score was derived by summing individual scores and converting them into predicted survival probabilities. Patients in the top 30% of total scores were defined as high risk. To facilitate individualized survival prediction for different patients, an interactive web-based dynamic nomogram was constructed.

#### 2.5.3 Differential gene analysis

Using the same inclusion criteria, EONDGC patients from the TCGA dataset were identified and scored via the nomogram. Patients were then classified into high- and low-risk groups. Differentially expressed genes (DEGs) analysis was performed between these groups to identify genes significantly upregulated in the high-risk group.

#### 2.5.4 Prognostic gene analysis

Genes with *P* < 0.1 in univariate Cox regression were included in multivariate analysis, and the model with the lowest AIC was selected. Genes with significant prognostic value were dichotomized into high- and low-expression groups according to the median transcripts per million (TPM) value or multi-gene expression ratio for Kaplan–Meier survival analysis. For immunophenoscore (IPS) analysis, immune checkpoint-related genes, including *PDCD1* and *CTLA4*, were included. IPS was calculated based on the gene expression profile using the method established in prior studies, reflecting the immune microenvironment’s response to immune checkpoint inhibition. The log_2_-transformed multi-gene TPM ratio was ultimately used as a molecular indicator.

### 2.6 *In vitro* validation

#### 2.6.1 Cell culture and transfection

Human gastric cancer cell lines MGC-803 and AGS were maintained at 37 °C with 5% CO_2_ in RPMI-1640 supplemented with 10% fetal bovine serum and penicillin–streptomycin. When cells reached 60–80% confluence, a transient transfection was performed using an *ARSB* overexpression plasmid; blank and empty-vector groups served as controls. Medium was replaced 6–8 h after transfection, and assays were initiated 24–48 h post-transfection.

#### 2.6.2 RT-qPCR

Total RNA was extracted and reverse-transcribed to cDNA. Quantitative PCR (SYBR Green) targeted *ARSB*, with GAPDH as the internal control. Relative mRNA levels were calculated using the 2^−ΔΔCt method, and melt-curve analysis was used to verify specificity. Effective *ARSB* upregulation at the mRNA level was confirmed prior to functional assays.

#### 2.6.3 Wound-healing assay

Cells were seeded in 6-well plates and grown to ~90–100% confluence. A linear scratch was created with a sterile 200 µL tip, debris was removed with PBS, and serum-free RPMI-1640 was added. Images were captured at 0, 24, and 48 h under an inverted microscope, using the same fields when possible. Migration area (%) was quantified as (A_0_ − A_t)/A_0_ × 100, where A_0_ and A_t denote the scratch area at 0 h and time t, respectively (measured in ImageJ). Each condition included at least three independent experiments, with ≥3–5 random fields per well.

#### 2.6.4 Transwell migration assay

Migration was assessed using 24-well Transwell inserts (8-µm pores; uncoated, no Matrigel). Cells suspended in serum-free medium were added to the upper chamber, and complete medium containing 10% FBS was placed in the lower chamber as a chemoattractant. After ~24 h at 37 °C, non-migrated cells on the upper surface were removed; migrated cells on the lower surface were fixed with methanol and stained with 0.1% crystal violet, then counted under an inverted microscope in ≥5 random fields per well. Experiments were independently repeated three times.

## 3 Results

### 3.1 The prognosis is impacted by age in GC, not anatomical subsite

**Figure 1:**
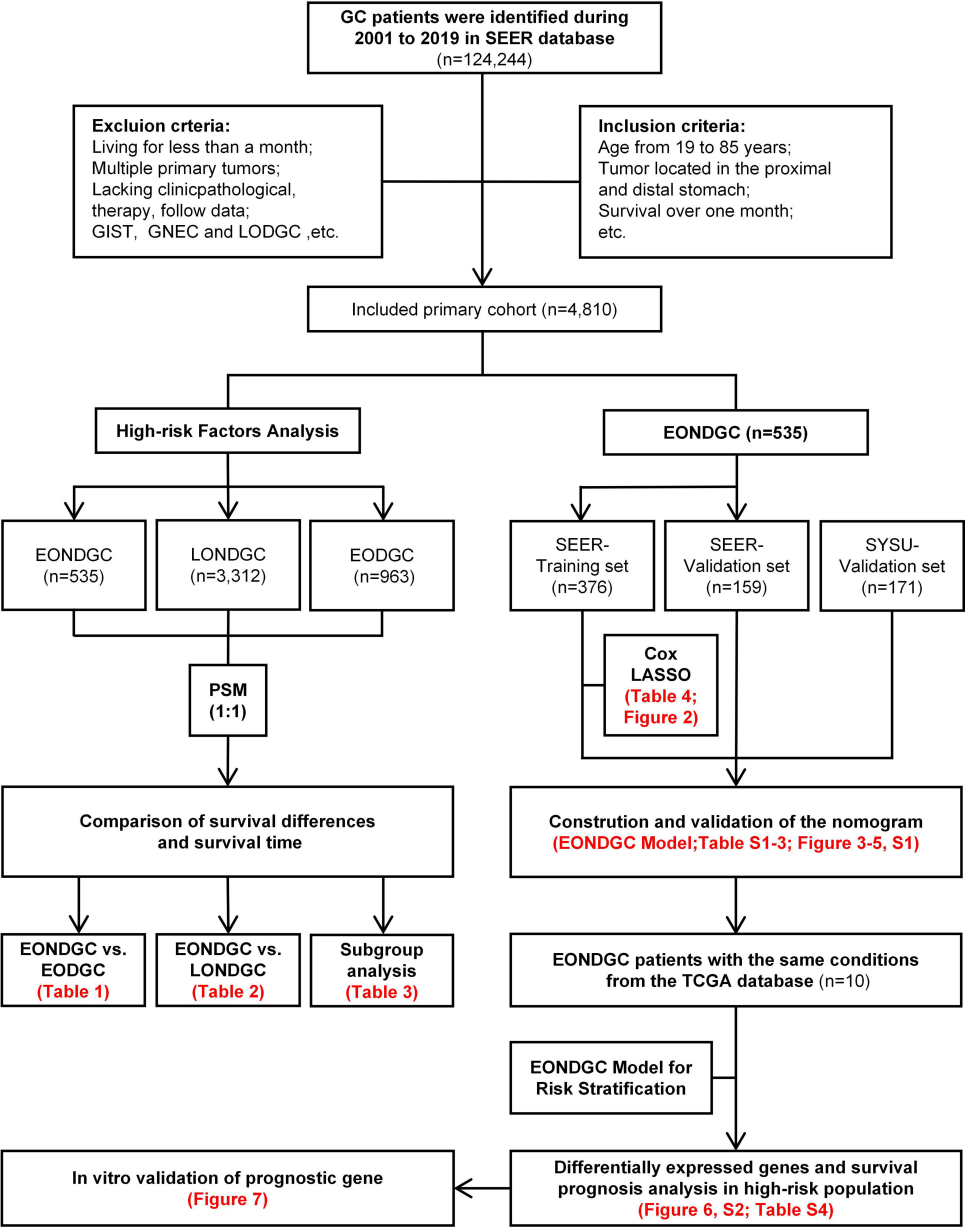
Flowchart of EONDGC prognostic model development, high-risk gene analysis, and *in-vitro* validation of *ARSB*.

The study was conducted according to the workflow illustrated in **Figure 1**. After screening and excluding 124,244 patients from the SEER database, 4,810 eligible cases were included: 535 with EONDGC, 3,312 with LONDGC, and 963 with EODGC.

Following PSM (**Table 1**), 264 patients remained in both the EONDGC and EODGC groups. Most baseline variables were well balanced, except for primary site (*P <* 0.001) and type of operation (*P <* 0.001), which remained significantly different. No significant differences were observed in vital status (14.4% *vs*. 16.3%, *P =* 0.618) or survival months (40.5 *vs*. 51.0, *P =* 0.629) after matching.

**Table 1:** Baseline characteristics of EONDGC and EODGC patients before and after PSM.

Characteristics	Before PSM		After PSM		Before	After
	EONDGC	EODGC	EONDGC	EODGC	*P* value	
	N=535	N=463	N=264	N=264		
Age of Diagnosis	44.0 [39.0;47.0]	43.0 [39.0;47.0]	44.0 [39.0;47.0]	43.0 [38.0;47.0]	0.023	0.672
Sex					<0.001	0.861
Female	174 (32.5%)	228 (49.2%)	119 (45.1%)	116 (43.9%)		
Male	361 (67.5%)	235 (50.8%)	145 (54.9%)	148 (56.1%)		
Race					<0.001	0.896
White	403 (75.3%)	250 (54.0%)	166 (62.9%)	161 (61.0%)		
Other	81 (15.1%)	123 (26.6%)	58 (22.0%)	60 (22.7%)		
Black	51 (9.53%)	90 (19.4%)	40 (15.2%)	43 (16.3%)		
Pathological Pattern					<0.001	0.642
Adenocarcinoma	401 (75.0%)	284 (61.3%)	181 (68.6%)	175 (66.3%)		
Signet ring	134 (25.0%)	179 (38.7%)	83 (31.4%)	89 (33.7%)		
Primary Site					<0.001	<0.001
Cardia	345 (64.5%)	0 (0.00%)	139 (52.7%)	0 (0.00%)		
Fundus	41 (7.66%)	0 (0.00%)	26 (9.85%)	0 (0.00%)		
Body	149 (27.9%)	0 (0.00%)	99 (37.5%)	0 (0.00%)		
Antrum	0 (0.00%)	396 (85.5%)	0 (0.00%)	224 (84.8%)		
Pylorus	0 (0.00%)	67 (14.5%)	0 (0.00%)	40 (15.2%)		
Pathological Grade					<0.001	0.086
Grade I	19 (3.55%)	10 (2.16%)	4 (1.52%)	9 (3.41%)		
Grade II	134 (25.0%)	49 (10.6%)	49 (18.6%)	35 (13.3%)		
Grade III	367 (68.6%)	388 (83.8%)	198 (75.0%)	213 (80.7%)		
Grade IV	15 (2.80%)	16 (3.46%)	13 (4.92%)	7 (2.65%)		
AJCC Stage					0.005	0.803
I	94 (17.6%)	105 (22.7%)	46 (17.4%)	54 (20.5%)		
II	140 (26.2%)	94 (20.3%)	63 (23.9%)	57 (21.6%)		
III	211 (39.4%)	159 (34.3%)	101 (38.3%)	98 (37.1%)		
IV	90 (16.8%)	105 (22.7%)	54 (20.5%)	55 (20.8%)		
T Stage					<0.001	0.139
T1	70 (13.1%)	75 (16.2%)	31 (11.7%)	42 (15.9%)		
T2	158 (29.5%)	130 (28.1%)	75 (28.4%)	76 (28.8%)		
T3	229 (42.8%)	138 (29.8%)	109 (41.3%)	86 (32.6%)		
T4	78 (14.6%)	120 (25.9%)	49 (18.6%)	60 (22.7%)		
N Stage					<0.001	0.986
N0	148 (27.7%)	127 (27.4%)	73 (27.7%)	74 (28.0%)		
N1	192 (35.9%)	117 (25.3%)	75 (28.4%)	75 (28.4%)		
N2	127 (23.7%)	120 (25.9%)	73 (27.7%)	75 (28.4%)		
N3	68 (12.7%)	99 (21.4%)	43 (16.3%)	40 (15.2%)		
M Stage					0.017	0.901
M0	475 (88.8%)	386 (83.4%)	225 (85.2%)	227 (86.0%)		
M1	60 (11.2%)	77 (16.6%)	39 (14.8%)	37 (14.0%)		
Tumor Size (mm)	40.0 [25.0;60.0]	45.0 [30.0;65.0]	41.0 [29.8;65.0]	45.0 [25.8;65.0]	0.012	0.64
LNR	0.12 [0.00;0.40]	0.23 [0.00;0.54]	0.16 [0.00;0.44]	0.20 [0.00;0.48]	<0.001	0.529
Type of Operation					<0.001	<0.001
Partial Gastrectomy	247 (46.2%)	297 (64.1%)	127 (48.1%)	155 (58.7%)		
Total Gastrectomy	154 (28.8%)	37 (7.99%)	93 (35.2%)	20 (7.58%)		
Gastrectomy(NOS)	134 (25.0%)	129 (27.9%)	44 (16.7%)	89 (33.7%)		
Radiation					0.043	0.727
No	250 (46.7%)	247 (53.3%)	143 (54.2%)	138 (52.3%)		
Yes	285 (53.3%)	216 (46.7%)	121 (45.8%)	126 (47.7%)		
Chemotherapy					0.002	0.919
No	105 (19.6%)	130 (28.1%)	64 (24.2%)	62 (23.5%)		
Yes	430 (80.4%)	333 (71.9%)	200 (75.8%)	202 (76.5%)		
Marital Status					0.99	0.928
Unmarried	185 (34.6%)	159 (34.3%)	93 (35.2%)	95 (36.0%)		
Married	350 (65.4%)	304 (65.7%)	171 (64.8%)	169 (64.0%)		
Household Income					0.748	0.725
≤$69,999	309 (57.8%)	273 (59.0%)	150 (56.8%)	155 (58.7%)		
>$70,000	226 (42.2%)	190 (41.0%)	114 (43.2%)	109 (41.3%)		
Survival Months	41.0 [18.0;92.5]	44.0 [14.0;102]	40.5 [16.0;100]	51.0 [15.0;104]	0.724	0.618
Vital Status					0.436	0.629
Alive	225 (42.1%)	207 (44.7%)	226 (85.6%)	221 (83.7%)		
Dead	310 (57.9%)	256 (55.3%)	38 (14.4%)	43 (16.3%)		

PSM was performed to balance all covariates except for primary site and type of operation, which were predetermined based on anatomical and treatment-related considerations. “Other” race refers to Asian or Pacific Islander. Pathological Grade I indicates well-differentiated tumors; Grade II, moderately differentiated; Grade III, poorly differentiated; and Grade IV, undifferentiated. The lymph node ratio (LNR) is defined as the number of positive lymph nodes divided by the total number examined. “Not otherwise specified (NOS)” is a classification used in the SEER database when the procedure type is not further specified. A *P* value < 0.05 was considered statistically significant.

GC cases were identified from the SEER database (2001-2019) under predefined inclusion/exclusion criteria, yielding a primary cohort (n=4,810). The left branch presents high-risk factor analyses: EONDGC (n=535) was propensity-matched 1:1 to LONDGC and to EODGC, followed by survival comparisons and subgroup analyses within EONDGC. The right branch shows model development within EONDGC: SEER cases were split into training (n=376) and internal-validation (n=159) sets, with an external SYSU cohort (n=171) for validation; variables were selected by Cox and LASSO and integrated into a prognostic nomogram. Using TCGA cases meeting the same conditions (n=10), risk scores were calculated to define a high-risk group, from which differentially expressed genes were identified and evaluated for prognostic value. *ARSB*, a candidate from this screen, underwent *in-vitro* validation by RT-qPCR, scratch (migration area at 24/48 h), and Transwell migration assays.

In the comparison between the EONDGC and LONDGC groups (**Table 2**), each group included 506 matched patients. There were significant differences in survival months (41.0 *vs*. 30.0, *P <* 0.001) and vital status (57.9% *vs*. 65.8%, *P =* 0.001). These findings underscore the prognostic distinctions between early- and late-onset, as well as distal and nondistal gastric cancer. To further explore the heterogeneity within EONDGC, we next performed subgroup analyses based on anatomical subsites.

**Table 2:** Baseline characteristics of EONDGC and LONDGC patients before and after PSM.

Characteristics	Before PSM		After PSM		Before	After
	EONDGC	LONDGC	EONDGC	LONDGC	*P* value	
	N=535	N=3312	N=506	N=506		
Age of Diagnosis	44.0 [39.0;47.0]	65.0 [58.0;72.0]	45.0 [39.2;47.0]	64.0 [58.0;72.0]	<0.001	<0.001
Sex					0.003	0.542
Female	174 (32.5%)	872 (26.3%)	154 (30.4%)	164 (32.4%)		
Male	361 (67.5%)	2440 (73.7%)	352 (69.6%)	342 (67.6%)		
Race					0.048	0.946
White	403 (75.3%)	2627 (79.3%)	387 (76.5%)	389 (76.9%)		
Other	81 (15.1%)	458 (13.8%)	72 (14.2%)	73 (14.4%)		
Black	51 (9.53%)	227 (6.85%)	47 (9.29%)	44 (8.70%)		
Pathological Pattern					<0.001	0.705
Adenocarcinoma	401 (75.0%)	2840 (85.7%)	397 (78.5%)	391 (77.3%)		
Signet ring	134 (25.0%)	472 (14.3%)	109 (21.5%)	115 (22.7%)		
Primary Site					0.033	0.965
Cardia	345 (64.5%)	2312 (69.8%)	337 (66.6%)	337 (66.6%)		
Fundus	41 (7.66%)	243 (7.34%)	35 (6.92%)	37 (7.31%)		
Body	149 (27.9%)	757 (22.9%)	134 (26.5%)	132 (26.1%)		
Pathological Grade					0.03	0.795
Grade I	19 (3.55%)	142 (4.29%)	19 (3.75%)	19 (3.75%)		
Grade II	134 (25.0%)	1021 (30.8%)	134 (26.5%)	135 (26.7%)		
Grade III	367 (68.6%)	2075 (62.7%)	338 (66.8%)	342 (67.6%)		
Grade IV	15 (2.80%)	74 (2.23%)	15 (2.96%)	10 (1.98%)		
AJCC Stage					0.001	0.994
I	94 (17.6%)	798 (24.1%)	90 (17.8%)	91 (18.0%)		
II	140 (26.2%)	849 (25.6%)	134 (26.5%)	132 (26.1%)		
III	211 (39.4%)	1253 (37.8%)	201 (39.7%)	199 (39.3%)		
IV	90 (16.8%)	412 (12.4%)	81 (16.0%)	84 (16.6%)		
T Stage					0.002	0.917
T1	70 (13.1%)	610 (18.4%)	68 (13.4%)	69 (13.6%)		
T2	158 (29.5%)	1046 (31.6%)	151 (29.8%)	160 (31.6%)		
T3	229 (42.8%)	1287 (38.9%)	215 (42.5%)	210 (41.5%)		
T4	78 (14.6%)	369 (11.1%)	72 (14.2%)	67 (13.2%)		
N Stage					0.01	0.477
N0	148 (27.7%)	1066 (32.2%)	140 (27.7%)	129 (25.5%)		
N1	192 (35.9%)	1248 (37.7%)	182 (36.0%)	204 (40.3%)		
N2	127 (23.7%)	598 (18.1%)	118 (23.3%)	105 (20.8%)		
N3	68 (12.7%)	400 (12.1%)	66 (13.0%)	68 (13.4%)		
M Stage					0.014	0.611
M0	475 (88.8%)	3049 (92.1%)	455 (89.9%)	449 (88.7%)		
M1	60 (11.2%)	263 (7.94%)	51 (10.1%)	57 (11.3%)		
Tumor Size (mm)	40.0 [25.0;60.0]	40.0 [25.0;60.0]	40.0 [27.0;60.0]	40.0 [25.0;60.0]	0.905	0.862
LNR	0.12 [0.00;0.40]	0.09 [0.00;0.37]	0.13 [0.00;0.40]	0.11 [0.00;0.41]	0.036	0.898
Type of Operation					0.184	0.731
Partial Gastrectomy	247 (46.2%)	1628 (49.2%)	238 (47.0%)	226 (44.7%)		
Total Gastrectomy	154 (28.8%)	831 (25.1%)	140 (27.7%)	149 (29.4%)		
Gastrectomy(NOS)	134 (25.0%)	853 (25.8%)	128 (25.3%)	131 (25.9%)		
Radiation					0.003	0.705
No	250 (46.7%)	1784 (53.9%)	232 (45.8%)	239 (47.2%)		
Yes	285 (53.3%)	1528 (46.1%)	274 (54.2%)	267 (52.8%)		
Chemotherapy					<0.001	0.445
No	105 (19.6%)	1172 (35.4%)	104 (20.6%)	115 (22.7%)		
Yes	430 (80.4%)	2140 (64.6%)	402 (79.4%)	391 (77.3%)		
Marital Status					0.01	0.841
Unmarried	185 (34.6%)	961 (29.0%)	168 (33.2%)	164 (32.4%)		
Married	350 (65.4%)	2351 (71.0%)	338 (66.8%)	342 (67.6%)		
Household Income					1	0.566
≤$69,999	309 (57.8%)	1914 (57.8%)	290 (57.3%)	300 (59.3%)		
>$70,000	226 (42.2%)	1398 (42.2%)	216 (42.7%)	206 (40.7%)		
Survival Months	41.0 [18.0;92.5]	32.0 [13.0;77.0]	41.0 [19.0;93.0]	30.0 [13.0;73.0]	<0.001	<0.001
Vital Status					0.009	0.010
Alive	225 (42.1%)	1196 (36.1%)	214 (42.3%)	173 (34.2%)		
Dead	310 (57.9%)	2116 (63.9%)	292 (57.7%)	333 (65.8%)		

PSM was conducted to balance all covariates except for age of diagnosis, which was predefined as the grouping variable. “Other” race refers to Asian or Pacific Islander. Pathological Grade I indicates well-differentiated tumors; Grade II, moderately differentiated; Grade III, poorly differentiated; and Grade IV, undifferentiated. The lymph node ratio (LNR) is defined as the number of positive lymph nodes divided by the total number examined. “Not otherwise specified (NOS)” is a classification used in the SEER database when the procedure type is not further specified. A *P* value < 0.05 was considered statistically significant.

In the subgroup analyses (**Table 3**), significant differences remained in the pathological pattern (*P =* 0.012), pathological grade (*P <* 0.001), and American Joint Committee on Cancer (AJCC) stage (*P =* 0.020) after matching. However, the prognostic differences were not statistically significant: the median survival months were 40.0 and 43.0 (*P =* 0.909), with mortality rates of 59.4% and 51.9% (*P =* 0.333), respectively.

**Table 3:** Subgroup analysis of EONDGC patients with tumors located in the Cardia versus Fundus/Body, before and after PSM.

Characteristics	Before PSM		After PSM		Before	After
	Cardia	Fundus/Body	Cardia	Fundus/Body	*P* value	
	N = 345	N = 190	N=106	N=106		
Age of Diagnosis	45.0 [39.0;48.0]	44.0 [39.2;47.0]	45.0 [38.2;47.0]	44.0 [40.0;47.0]	0.151	0.932
Sex					<0.001	1
Female	77 (22.3%)	97 (51.1%)	32 (30.2%)	33 (31.1%)		
Male	268 (77.7%)	93 (48.9%)	74 (69.8%)	73 (68.9%)		
Race					<0.001	0.694
White	297 (86.1%)	106 (55.8%)	83 (78.3%)	82 (77.4%)		
Other	32 (9.28%)	49 (25.8%)	17 (16.0%)	15 (14.2%)		
Black	16 (4.64%)	35 (18.4%)	6 (5.66%)	9 (8.49%)		
Pathological Pattern					<0.001	0.012
Adenocarcinoma	282 (81.7%)	119 (62.6%)	87 (82.1%)	70 (66.0%)		
Signet ring	63 (18.3%)	71 (37.4%)	19 (17.9%)	36 (34.0%)		
Pathological Grade					<0.001	<0.001
Grade I	16 (4.64%)	3 (1.58%)	4 (3.77%)	2 (1.89%)		
Grade II	117 (33.9%)	17 (8.95%)	33 (31.1%)	9 (8.49%)		
Grade III	204 (59.1%)	163 (85.8%)	67 (63.2%)	92 (86.8%)		
Grade IV	8 (2.32%)	7 (3.68%)	2 (1.89%)	3 (2.83%)		
AJCC Stage					0.05	0.02
I	57 (16.5%)	37 (19.5%)	15 (14.2%)	20 (18.9%)		
II	82 (23.8%)	58 (30.5%)	25 (23.6%)	37 (34.9%)		
III	151 (43.8%)	60 (31.6%)	51 (48.1%)	29 (27.4%)		
IV	55 (15.9%)	35 (18.4%)	15 (14.2%)	20 (18.9%)		
T Stage					<0.001	0.007
T1	46 (13.3%)	24 (12.6%)	13 (12.3%)	14 (13.2%)		
T2	101 (29.3%)	57 (30.0%)	29 (27.4%)	36 (34.0%)		
T3	164 (47.5%)	65 (34.2%)	54 (50.9%)	32 (30.2%)		
T4	34 (9.86%)	44 (23.2%)	10 (9.43%)	24 (22.6%)		
N Stage					0.006	0.005
N0	88 (25.5%)	60 (31.6%)	24 (22.6%)	29 (27.4%)		
N1	131 (38.0%)	61 (32.1%)	39 (36.8%)	41 (38.7%)		
N2	92 (26.7%)	35 (18.4%)	32 (30.2%)	13 (12.3%)		
N3	34 (9.86%)	34 (17.9%)	11 (10.4%)	23 (21.7%)		
M Stage					0.019	0.077
M0	315 (91.3%)	160 (84.2%)	99 (93.4%)	90 (84.9%)		
M1	30 (8.70%)	30 (15.8%)	7 (6.60%)	16 (15.1%)		
Tumor Size (mm)	40.0 [26.0;60.0]	40.0 [25.0;68.8]	40.5 [25.5;57.8]	40.5 [30.0;70.0]	0.15	0.189
LNR	0.11 [0.00;0.33]	0.14 [0.00;0.49]	0.13 [0.00;0.32]	0.15 [0.00;0.52]	0.128	0.26
Type of Operation					0.002	0.181
Partial Gastrectomy	177 (51.3%)	70 (36.8%)	51 (48.1%)	38 (35.8%)		
Total Gastrectomy	84 (24.3%)	70 (36.8%)	28 (26.4%)	37 (34.9%)		
Gastrectomy(NOS)	84 (24.3%)	50 (26.3%)	27 (25.5%)	31 (29.2%)		
Radiation					<0.001	<0.001
No	127 (36.8%)	123 (64.7%)	32 (30.2%)	62 (58.5%)		
Yes	218 (63.2%)	67 (35.3%)	74 (69.8%)	44 (41.5%)		
Chemotherapy					0.465	0.611
No	64 (18.6%)	41 (21.6%)	20 (18.9%)	24 (22.6%)		
Yes	281 (81.4%)	149 (78.4%)	86 (81.1%)	82 (77.4%)		
Marital Status					0.82	0.313
Unmarried	121 (35.1%)	64 (33.7%)	33 (31.1%)	41 (38.7%)		
Married	224 (64.9%)	126 (66.3%)	73 (68.9%)	65 (61.3%)		
Household Income					0.965	0.268
≤$69,999	200 (58.0%)	109 (57.4%)	18 (17.0%)	23 (21.7%)		
>$70,000	145 (42.0%)	81 (42.6%)	88 (83.0%)	83 (78.3%)		
Survival Months	38.0 [19.0;92.0]	43.0 [18.0;93.0]	40.0 [17.0;92.0]	43.0 [19.0;94.5]	0.956	0.909
Vital Status					0.034	0.333
Alive	133 (38.6%)	92 (48.4%)	43 (40.6%)	51 (48.1%)		
Dead	212 (61.4%)	98 (51.6%)	63 (59.4%)	55 (51.9%)		

PSM was performed to match for race, sex, age of diagnosis, marital status, and household income. “Other” race refers to Asian or Pacific Islander. Pathological Grade I indicates well-differentiated tumors; Grade II, moderately differentiated; Grade III, poorly differentiated; and Grade IV, undifferentiated. The lymph node ratio (LNR) is defined as the number of positive lymph nodes divided by the total number examined. “Not otherwise specified (NOS)” is a classification used in the SEER database when the procedure type is not further specified. A *P* value < 0.05 was considered statistically significant.

After confirming that anatomical subsites within the EONDGC did not significantly influence prognosis, we proceeded with model development. A statistical comparison of the baseline variables between the SEER-training set (n = 376) and SEER-validation set (n = 159) was conducted (**Supplementary Table S1**), confirming that their distributions were not significantly different. Baseline characteristics of the SEER-training cohort and the SYSU-validation cohort were summarized in **Supplementary Table S2**.

### 3.2 The EONDGC prognostic model is composed of seven key factors

Univariate and multivariate Cox proportional hazards regression analyses were conducted to identify prognostic factors associated with overall survival (OS) in patients with EONDGC (**Table 4**).

**Table 4:** Univariate and multivariate Cox regression analysis of overall survival in the SEER-training set.

Characteristics	Univariate Analysis			Multivariate Analysis		
	HR	95% CI	*P* value	HR	95% CI	*P* value
Age of Diagnosis	0.99	0.97-1.01	0.213			
Sex						
Female	Reference					
Male	0.89	0.67-1.18	0.413			
Race						
White	Reference			Reference		
Other	0.62	0.41-0.94	0.026	0.59	0.38-0.92	0.019
Black	1.39	0.93-2.09	0.112	0.83	0.54-1.28	0.395
Pathological Pattern						
Adenocarcinoma	Reference					
Signet ring	1.1	0.82-1.48	0.532			
Primary Site						
Cardia	Reference					
Fundus	0.8	0.46-1.39	0.435			
Body	0.95	0.70-1.28	0.731			
Pathological Grade						
Grade I	Reference			Reference		
Grade II	2.96	0.92-9.57	0.07	2	0.60-6.60	0.256
Grade III	5.08	1.62-15.92	0.005	2.66	0.83-8.52	0.101
Grade IV	12.93	3.42-48.97	<0.001	5.33	1.33-21.25	0.018
AJCC Stage						
I	Reference					
II	3.54	1.88-6.66	<0.001			
III	6.24	3.14-11.39	<0.001			
IV	14.51	7.75-27.19	<0.001			
T Stage						
T1	Reference			Reference		
T2	5.1	2.54-10.25	<0.001	2.58	1.2-5.55	0.016
T3	5.9	2.98-11.69	<0.001	3.22	1.48-7.04	0.003
T4	8.13	3.95-16.71	<0.001	4.64	2.05-10.52	<0.001
N Stage						
N0	Reference			Reference		
N1	3.65	2.32-5.73	<0.001	2.15	1.29-3.57	0.003
N2	6.62	4.18-10.46	<0.001	2.39	1.38-4.12	0.002
N3	7.49	4.52-12.42	<0.001	2.42	1.31-4.48	0.005
M Stage						
M0	Reference			Reference		
M1	3.83	2.71-5.42	<0.001	2.56	1.78-3.69	<0.001
Tumor Size (mm)	1.01	1.01-1.01	<0.001			
LNR	9.58	6.44-14.25	<0.001	4.03	2.35-6.89	<0.001
Type of Operation						
Partial Gastrectomy	Reference					
Total Gastrectomy	1.04	0.76-1.42	0.817			
Gastrectomy(NOS)	0.84	0.6-1.18	0.315			
Radiation						
No	Reference					
Yes	1.01	0.77-1.32	0.95			
Chemotherapy						
No	Reference			Reference		
Yes	1.84	1.26-2.68	0.002	0.5	0.33-0.77	0.002
Marital Status						
Unmarried	Reference			Reference		
Married	0.72	0.55-0.94	0.017	0.74	0.56-0.98	0.037
Household Income						
≤$69,999	Reference					
>$70,000	0.87	0.66-1.14	0.304			

Hazard ratios (HRs) and 95% confidence intervals (CIs) were calculated using Cox proportional hazards models. Variables with *P* < 0.1 in univariate analysis—including Race, Pathological Grade, AJCC Stage, T Stage, N Stage, M Stage, Tumor Size, lymph node ratio (LNR), Chemotherapy, and Marital Status—were entered into the multivariate analysis, in which the model with the lowest AIC was selected as the final result. “Other” race refers to Asian or Pacific Islander. Pathological Grade I indicates well-differentiated tumors; Grade II, moderately differentiated; Grade III, poorly differentiated; and Grade IV, undifferentiated. The LNR is defined as the number of positive lymph nodes divided by the total number examined. “Not otherwise specified (NOS)” is a classification used in the SEER database when the procedure type is not further specified. A *P* value < 0.05 was considered statistically significant.

In the univariate analysis, poor pathological grade (Grade IV: hazard ratio [HR] = 12.93, 95% confidence interval [CI]: 3.42–48.97, *P* < 0.001), AJCC stage IV (HR = 14.51, 95% CI: 7.75–27.19, *P* < 0.001), T4 stage (HR = 8.13, 95% CI: 3.95–16.71, *P* < 0.001), N3 stage (HR = 7.49, 95% CI: 4.52–12.42, *P* < 0.001), M1 stage (HR = 3.83, 95% CI: 2.71–5.42, *P* < 0.001), and a high lymph node ratio (LNR; HR = 9.58, 95% CI: 6.44–14.25, *P* < 0.001) were all significantly associated with worse prognosis. Additionally, larger tumor size (HR = 1.01, 95% CI: 1.01–1.01, *P* < 0.001) and receipt of chemotherapy (HR = 1.84, 95% CI: 1.26–2.68, *P* = 0.002) were also linked to increased mortality. Conversely, being of “Other” racial background (HR = 0.62, 95% CI: 0.41–0.93, *P* = 0.026) and being married (HR = 0.72, 95% CI: 0.55–0.94, *P* = 0.017) were associated with reduced mortality risk.

In the multivariate analysis, independent predictors of poor survival included T4 stage (HR = 4.64, 95% CI: 2.05–10.52, *P* < 0.001), N3 stage (HR = 2.42, 95% CI: 1.31–4.48, *P* = 0.005), M1 stage (HR = 2.56, 95% CI: 1.78–3.69, *P* < 0.001), and LNR (HR = 4.03, 95% CI: 2.35–6.89, *P* < 0.001). Meanwhile, chemotherapy was independently associated with improved survival (HR = 0.50, 95% CI: 0.33–0.77, *P* = 0.002), as were “Other” racial background (HR = 0.59, 95% CI: 0.38–0.92, *P* = 0.019) and married status (HR = 0.74, 95% CI: 0.56–0.98, *P* = 0.037).

**Figure 2:**
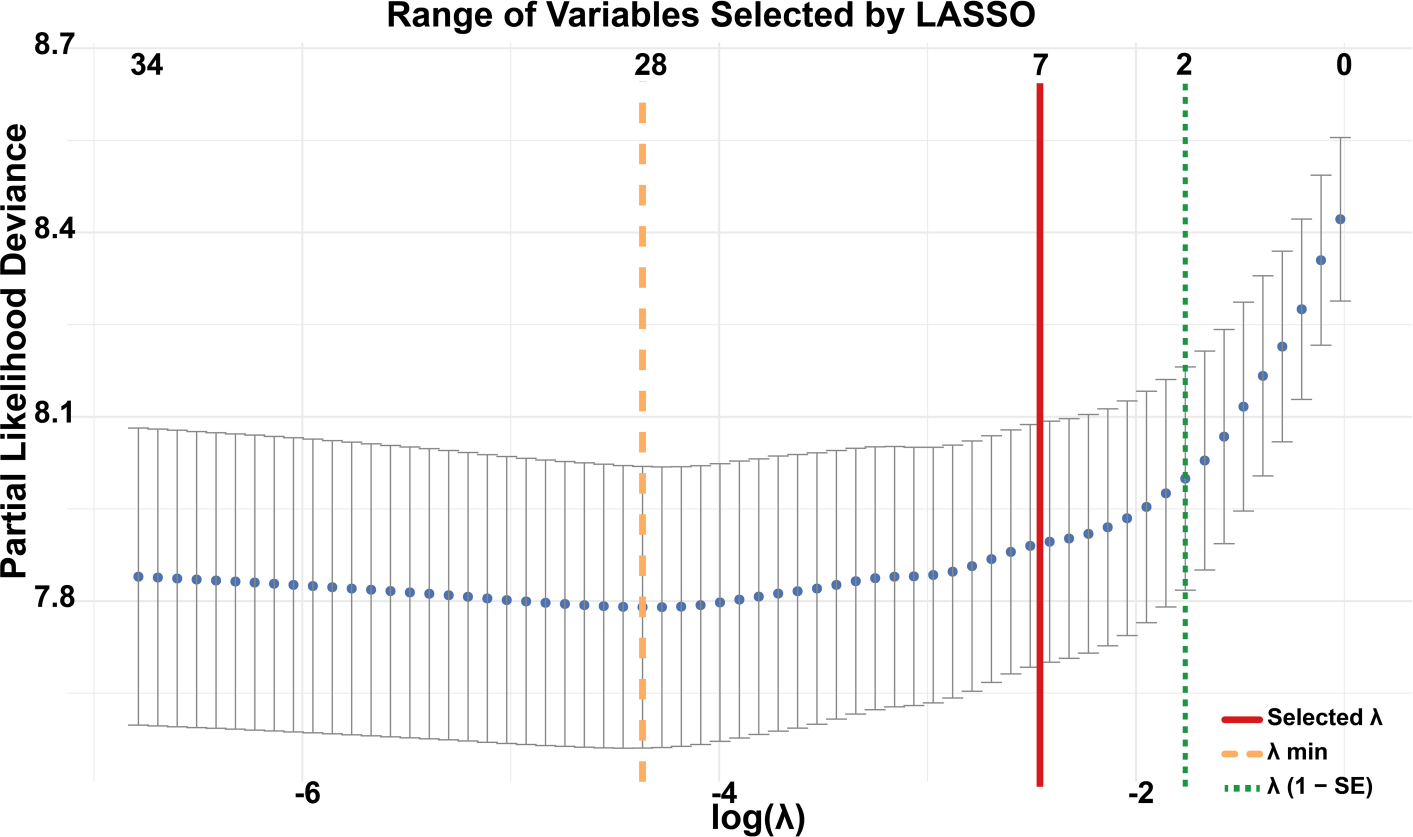
Variable selection using LASSO regression. The y-axis represents partial likelihood deviance from the Cox model, with each dot corresponding to a different log(λ) value in 10-fold cross-validation. The orange dashed line indicates the λ that minimizes the cross-validated error (λ min), selecting 28 variables. The green dotted line marks the largest λ within one standard error of the minimum (λ 1-SE), resulting in a parsimonious model with 2 variables. The red solid line represents the λ value ultimately chosen in this study, which identified 7 variables as the optimal trade-off between model performance and simplicity.

Given the large number of prognostic factors identified by Cox regression, we applied LASSO regression to reduce variable dimensionality and enhance the model’s predictive efficiency. LASSO regression showed a variable selection range of 2 to 28 predictors (**Figure 2**). Seven variables were ultimately retained on the basis of their prognostic significance: race, pathological grade, T stage, N stage, M stage, LNR, and chemotherapy.

**Figure 3:**
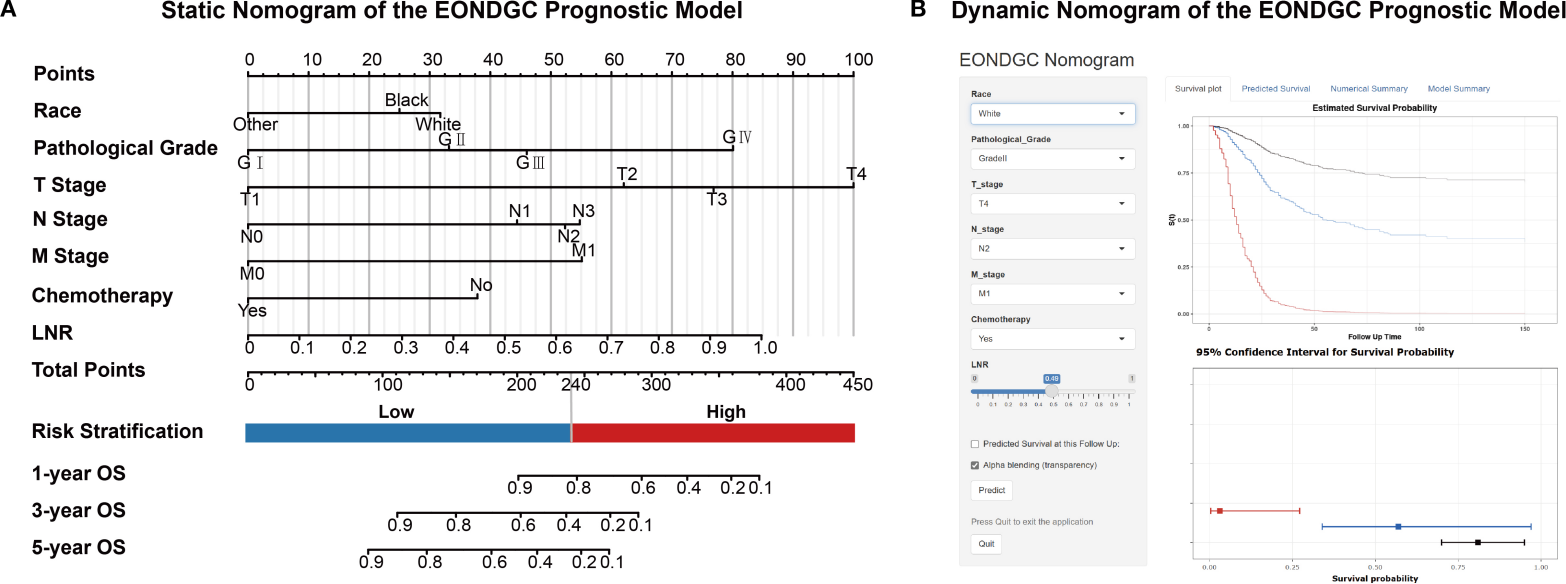
Static and dynamic nomograms for the EONDGC prognostic model. **(A)** Static nomogram constructed based on multivariable Cox and LASSO regression. Each variable contributes a specific point value reflecting its prognostic weight. The total point score is used to estimate 1-, 3-, and 5-year OS probabilities. A risk stratification threshold of 240 points is used to divide patients into high- and low-risk groups, supporting individualized prognostic evaluation. **(B)** Interactive dynamic nomogram interface. Users can freely select combinations of seven prognostic variables from the input panel on the left. Upon clicking the “Predict” button, individualized survival predictions are displayed, including Kaplan–Meier survival curves and corresponding 95% CI, enabling real-time visualization of personalized survival probability (https://zhangzhq79sysu.shinyapps.io/EONDGC/).

These variables were integrated into a nomogram to visualize the EONDGC prognostic model (**Figure 3A**). In the nomogram, each variable was assigned a point value (**Supplementary Table S3**), and the total score was calculated by summing across variables. This total risk score was then mapped to predict 1-, 3-, and 5-year OS probabilities. Furthermore, an interactive web-based dynamic nomogram was developed, allowing users to input specific clinical information and to obtain individualized survival predictions (https://zhangzhq79sysu.shinyapps.io/EONDGC/, **Figure 3B**).

### 3.3 The model performance outperforms TNM staging system

**Figure 4:**
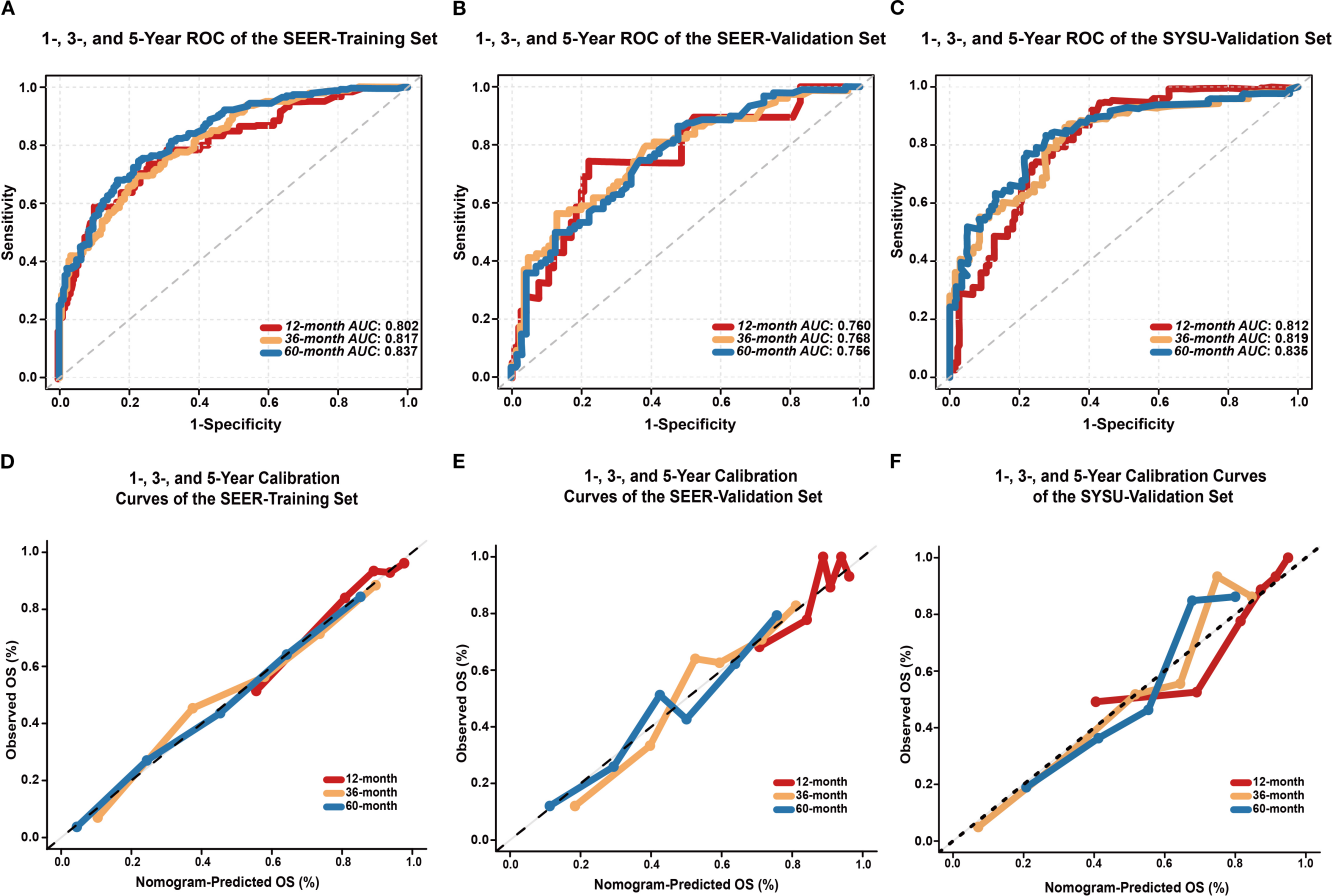
Performance evaluation of the EONDGC prognostic nomogram. **(A)** Time-dependent ROC curves of the SEER-training set. The model demonstrated strong discriminatory power with AUC values of 0.802, 0.817, and 0.837 for 1-, 3-, and 5-year OS, respectively. AUC values greater than 0.75 generally indicate good discriminative ability of the model. **(B)** ROC curves of the SEER-validation set. The model maintained satisfactory predictive performance in the external cohort, with AUCs of 0.760, 0.768, and 0.756 at 1, 3, and 5 years, respectively. **(C)** In the external validation cohort from SYSU, the model exhibited excellent predictive ability, with AUCs of 0.812, 0.819, and 0.836 at 1, 3, and 5 years, respectively. **(D)** Calibration curves of the SEER-training set at 12, 36, and 60 months show excellent agreement between nomogram-predicted and observed OS probabilities. **(E)** Calibration curves of the SEER-validation set also demonstrate good consistency. **(F)** In the external validation cohort from SYSU, calibration curves at 1, 3, and 5 years similarly showed good concordance between predicted and actual OS. In all sets, the curves closely follow the 45° diagonal line, indicating accurate survival prediction and good model calibration. A curve that aligns with the diagonal reflects a minimal deviation between predicted and actual outcomes, which supports the reliability of the model.

The C-index was 0.758 (95% CI: 0.725–0.791) in the SEER-training set, 0.718 (95% CI: 0.663–0.773) in the SEER-validation set, and 0.762 (95% CI: 0.719–0.805) in the SYSU-validation set. In the SEER-training set (**Figure 4A**), the AUCs at 1, 3, and 5 years were 0.802 (95% CI: 0.738–0.866), 0.817 (95% CI: 0.769–0.854), and 0.837 (95% CI: 0.784–0.866), respectively. In the SEER-validation set (**Figure 4B**), the corresponding AUCs were 0.760 (95% CI: 0.632–0.877), 0.768 (95% CI: 0.682–0.834), and 0.756 (95% CI: 0.668–0.821). In the SYSU-validation set (**Figure 4C**), the model achieved AUCs of 0.812 (95% CI: 0.760–0.890) at 1 year, 0.819 (95% CI: 0.757–0.885) at 3 years, and 0.836 (95% CI: 0.761–0.886) at 5 years.

In both the SEER-training (**Figure 4D**) and SEER-validation sets (**Figure 4E**), the curves at all time points closely aligned with the ideal 45-degree reference line. In the SYSU-validation set (**Figure 4F**), the 1-, 3-, and 5-year calibration curves showed moderate deviation at intermediate predicted probabilities but remained close to the ideal line in the high-probability range.

To further evaluate the clinical decision-making value of the EONDGC model, we compared its net benefit with that of the traditional TNM staging system using DCA. As shown in **Supplementary Figures S1A-C** for the SEER-training set and **Supplementary Figures S1D-F** for the SEER-validation set, the EONDGC model consistently provided a wider range of clinical net benefit than did the traditional Tumor-Node-Metastasis (TNM) staging system across all time points. In the SYSU-validation set (**Supplementary Figures S1G-I**), the model exhibited higher net benefit than the TNM staging system at 1-, 3-, and 5-year time points across a wide range of threshold probabilities.

### 3.4 The model effectively differentiates high- and low-risk groups

**Figure 5:**
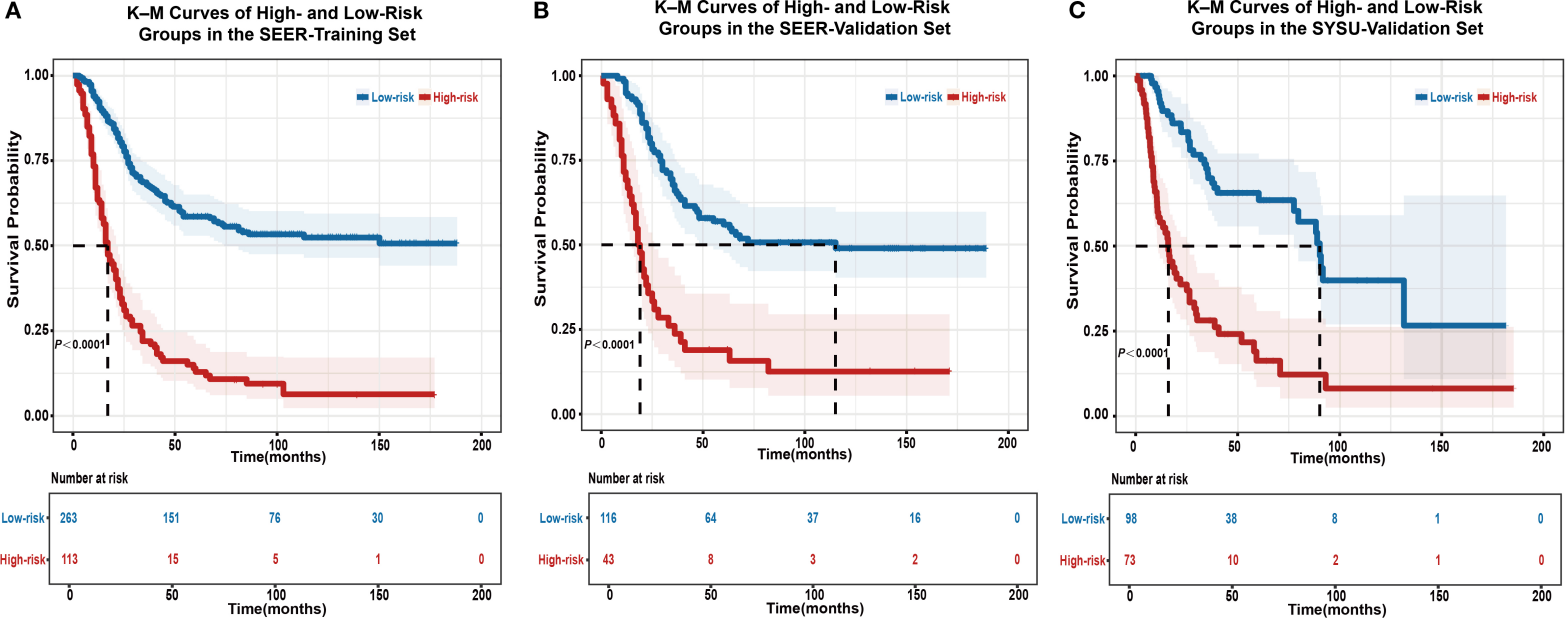
Kaplan–Meier survival analysis for risk stratification using the EONDGC model in the training and validation sets. **(A)** Kaplan–Meier curve of the SEER-training set shows that patients classified into the high-risk group had significantly worse OS than those in the low-risk group (*P* < 0.0001). **(B)** In the SEER-validation set, the model’s risk stratification was successfully reproduced, with a clear separation in survival curves between the two groups (*P* < 0.0001). **(C)** In the external validation cohort from SYSU, the high-risk group also showed significantly worse OS compared to the low-risk group (*P* < 0.0001), confirming the model’s prognostic stratification performance in a real-world clinical setting. Patients were divided based on a risk score cut-off derived from the nomogram. Shaded areas indicate 95% CI, and the number of patients at risk at each follow-up point is listed below the plots. These results highlight the model’s robust ability to distinguish prognosis and support its clinical applicability for individualized survival prediction. A *P* value < 0.05 was considered statistically significant.

Significant differences in OS were observed between the high- and low-risk groups in the SEER-training (**Figure 5A**), SEER-validation (**Figure 5B**), and SYSU-validation set (**Figure 5C**; *P* < 0.0001), indicating consistent and reproducible stratification performance of the model across internal and external datasets.

### 3.5 The ARSB-PDCD1 ratio stratifies immune-related prognostic risk in EONDGC

**Figure 6:**
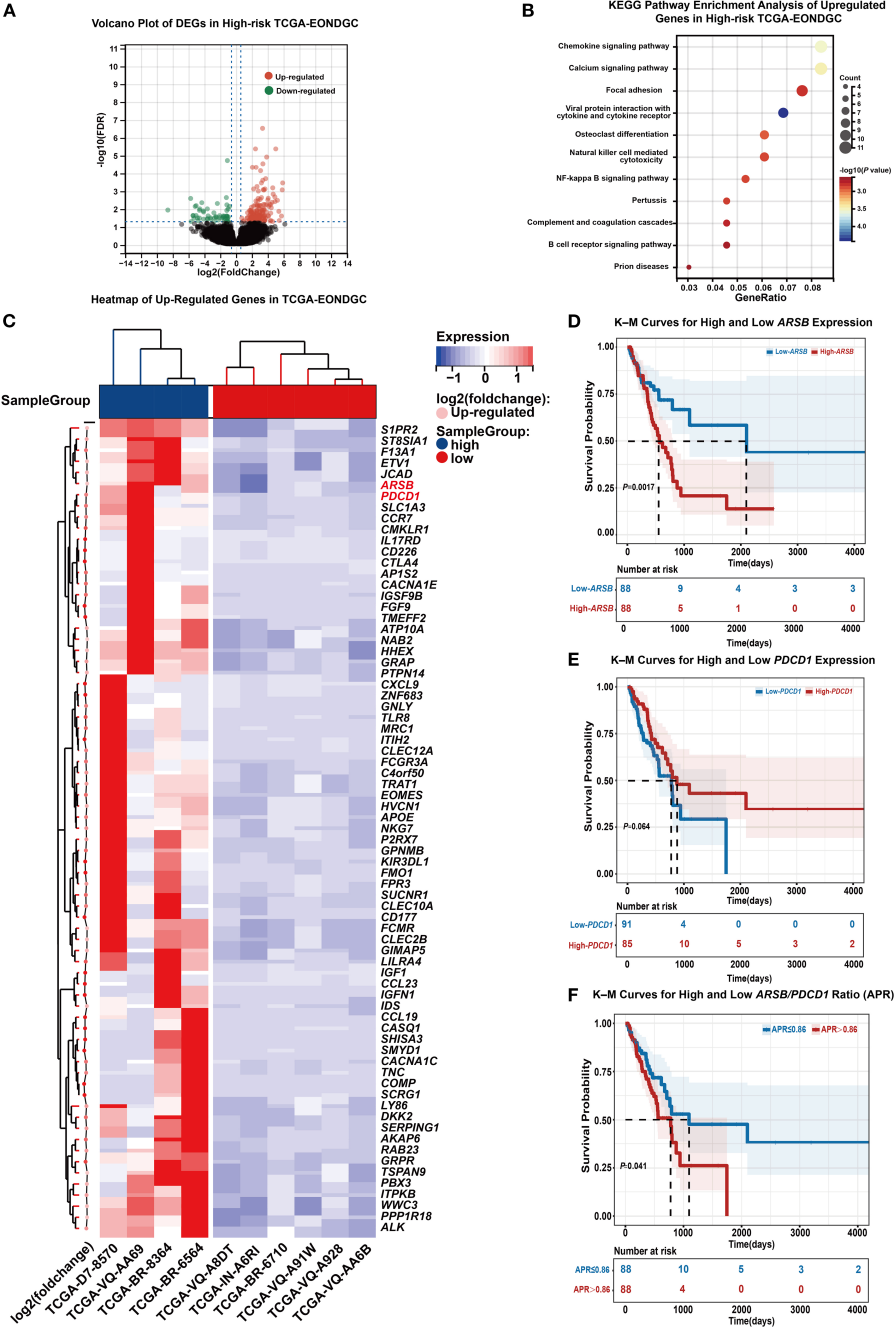
Integrated transcriptomic and survival analysis in high-risk EONDGC. **(A)** Volcano plot of DEGs between high- and low-risk EONDGC patients, defined by nomogram-derived risk scores in the TCGA cohort. Red dots indicate significantly upregulated genes, green dots indicate significantly downregulated genes, and black dots denote non-significant genes. **(B)** KEGG pathway enrichment analysis of upregulated genes in the high-risk group. Each bubble represents an enriched pathway; bubble size reflects the number of genes, and color indicates statistical significance (–log_10_P), with warmer colors corresponding to higher significance. Enriched pathways were mainly related to immune regulation and intracellular signaling. **(C)** Heatmap showing expression patterns of upregulated DEGs between high- and low-risk EONDGC samples. Rows represent individual genes, and columns represent patient samples. Red and blue indicate higher or lower expression relative to row-wise mean values. Hierarchical clustering reveals distinct expression profiles between risk groups. **(D-F)** Kaplan–Meier survival curves stratified by expression of *ARSB* **(D)**, *PDCD1* **(E)**, and the APR **(F)**. Patients were divided into high and low groups using the median value for each marker. High *ARSB* expression and elevated APR were significantly associated with worse overall survival (*P* = 0.0017 and *P* = 0.041, respectively), while *PDCD1* showed a non-significant trend toward improved prognosis (*P* = 0.064). Shaded areas represent 95% CI. Numbers at risk are shown below each time point. A P value < 0.05 was considered statistically significant.

A total of 10 EONDGC patients from the TCGA cohort were included. Using the EONDGC model, we calculated risk scores for each patient and identified those in the high-risk group for further molecular characterization. DEGs analysis in high-risk patients was visualized using a volcano plot (**Figure 6A**), identifying 73 significantly upregulated genes. These genes were subsequently subjected to KEGG pathway enrichment analysis (**Figure 6B**), and their expression profiles are displayed in a heatmap (**Figure 6C**). A global comparison of DEGs analysis between early-onset and late-onset NDGC was presented in **Supplementary Figures S2A, B**. Based on the upregulated genes (**Supplementary Table S4**), univariate and multivariate Cox regression analyses were performed in the TCGA-NDGC cohort to evaluate their prognostic significance.

In the univariate analysis, genes such as *JCAD* (HR = 2.02, 95% CI: 1.21–3.37, *P* = 0.007), *WWC3* (HR = 1.88, 95% CI: 1.14–3.12, *P* = 0.014), *TNC* (HR = 1.7, 95% CI: 1.04–2.79, *P* = 0.034), and *ARSB* (HR = 2.23, 95% CI: 1.33–3.72, *P* = 0.002) were associated with a greater risk of death. In the multivariate analysis, *ARSB* (HR = 2.40, 95% CI: 1.38–4.17, *P* = 0.002) and *PDCD1* (HR = 0.50, 95% CI: 0.27–0.91, *P* = 0.024) remained statistically significant. To further validate their prognostic stratification potential and explore their combined impact, we conducted Kaplan-Meier survival analyses based on *ARSB*, *PDCD1*, and their expression ratio.

As shown in **Figure 6D**, patients with high *ARSB* expression had significantly worse OS than those with low expression (18.7 *vs*. 70.0 months, *P <* 0.05). In **Figure 6E**, higher *PDCD1* expression was associated with a better prognosis, although the difference did not reach statistical significance (21.9 *vs*. 25.9 months, *P* = 0.064). **Figure 6F** showed that patients with an *ARSB/PDCD1* ratio (APR) >0.86 had significantly worse survival than those with an APR ≤ 0.86 (25.9 *vs*. 36.5 months, *P* = 0.041). In addition, APR was inversely correlated with the IPS (**Supplementary Figure S2C**). ROC analysis further showed that APR discriminated IPS status with an AUC of 0.729 (**Supplementary Figure S2D**).

### 3.6 ARSB overexpression enhances migratory capacity of GC cells

**Figure 7:**
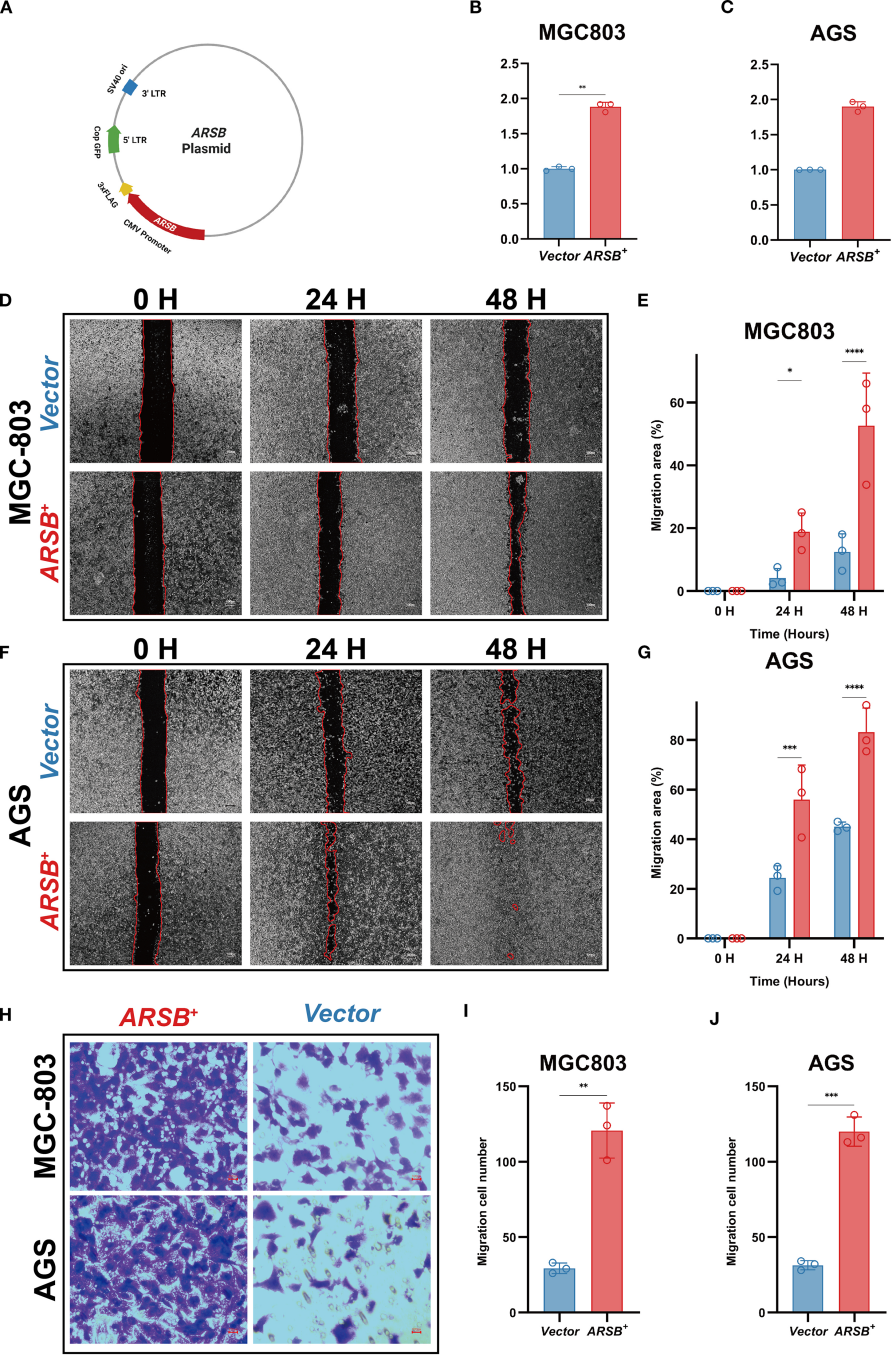
*In-vitro* validation of *ARSB* in GC cells. **(A)** Schematic of the *ARSB* overexpression plasmid. **(B, C)** RT-qPCR verifying *ARSB* mRNA upregulation (GAPDH as reference). **(D, E)** Representative images **(D)** and quantification **(E)** of the wound-healing assays in MGC803 cells at 0, 24, and 48 hours. **(F, G)** Representative images **(F)** and quantification **(G)** of the wound-healing assays in AGS cells at 0, 24, and 48 hours. **(H-J)** Representative images of Transwell migration assays **(H)** and quantification **(I-J)** of migrated cells per field in MGC803 **(I)** and AGS **(J)** cells. Data are mean ± SD; n = 3; two-sided tests; *P < 0.05, **P < 0.01, ***P < 0.001; scale bar = 100 μm.

To enable gain-of-function testing, an *ARSB* overexpression plasmid was constructed (**Figure 7A**) and transiently introduced into MGC-803 and AGS cells. RT-qPCR confirmed robust *ARSB* mRNA upregulation relative to empty-vector controls (**Figures 7B, C**). In wound-healing assays, representative images at 0/24/48 h are shown (**Figures 7D, F**), and quantification demonstrated significantly greater wound closure at 24 and 48 h in *ARSB*-overexpressing cells versus controls in both lines (**Figures 7E, G**). In Transwell migration assays using uncoated inserts, representative micrographs revealed more migrated cells upon *ARSB* overexpression (**Figure 7H**), with corresponding counts significantly increased for both MGC-803 and AGS (**Figures 7I, J**). Collectively, these results indicate that forced *ARSB* expression enhances the migratory capacity of GC cells *in vitro*.

## 4 Discussion

### 4.1 Study overview and significance

This study analyzed EONDGC using the SEER database, comparing it with LONDGC and EODGC, alongside subgroup analyses within EONDGC. We found that tumor location did not significantly affect survival in early-onset cases, whereas age was a key prognostic factor, suggesting location influences clinical presentation more than survival. By integrating clinical and molecular factors, this study pioneers a prognostic model for EONDGC, introducing the novel APR index to enhance risk stratification.

### 4.2 Clinical prognostic model

Unlike most previous models for young or early-onset GC, which were primarily based on TNM staging, tumor size, or location (26, 27), our EONDGC model incorporates seven clinically relevant variables, including LNR, race, and chemotherapy. LNR has been shown to outperform conventional N staging, with reported C-index improvements from 0.665 to 0.773 when included (28). Although a previous model included external validation, our study was specifically designed as a prognosis-oriented comparative analysis, ensuring clear clinical relevance and methodological rigor (26). This prognosis-driven approach, together with the inclusion of LNR, treatment factors, and multi-cohort validation, enhances the model’s methodological robustness and clinical applicability.

### 4.3 Molecular prognostic factors and APR index

To explore molecular mechanisms, we identified *ARSB* and *PDCD1* as independent prognostic biomarkers. *PDCD1*, a key immune checkpoint receptor, has been reported to correlate with favorable outcomes in immune-active GC, particularly when expressed on CD8^+^ T cells. However, such studies primarily reflect immune status without addressing tumor-intrinsic biology (29–31). In contrast, *ARSB* is a tumor-derived enzyme linked to Wnt/β-catenin signaling, upregulated in EONDGC and associated with poor prognosis (32, 33). To integrate these opposing effects, we proposed the APR, which effectively stratified survival risk. Unlike existing signatures that focus solely on immune or metabolic markers, APR provides a concise, biologically interpretable index reflecting tumor–immune interaction. To complement these clinical findings, we performed *in-vitro* gain-of-function assays in AGS and MGC-803 cells: transient *ARSB* overexpression accelerated wound closure at 24/48 h and increased Transwell migration counts, indicating that *ARSB* enhances gastric cancer cell motility and providing experimental support for its association with poorer survival in EONDGC.

### 4.4 Innovations and clinical implications

To the best of our knowledge, this study is the first to specifically investigate EONDGC, an understudied GC subtype with distinct clinical features. By separating this population, we identified prognostic patterns that enabled the development of a site-specific model outperforming conventional TNM staging. Furthermore, we propose the APR as a novel molecular biomarker. As the first index combining *ARSB* and *PDCD1* expression, APR shows promise for individualized treatment, particularly in immunotherapy, due to its relevance to the *PD-1/PD-L1* axis (30, 34). The concordant increase in migration readouts with *ARSB* overexpression suggests that APR may partly capture motility-associated risk, supporting its potential utility for refined surveillance and treatment planning.

### 4.5 Limitations and future directions

Despite these advances, several limitations should be acknowledged. First, as a retrospective study, our analysis is inherently subject to selection bias and potential misclassification resulting from incomplete clinical information—such as insufficient lymph node harvest—although PSM was applied to reduce observable confounding and approximate the conditions of a randomized controlled trial. Second, due to the limited number of EONDGC cases in TCGA (n = 10), NDGC cases (n = 176) were used for validation. This small sample size resulted from strict inclusion criteria and the requirement for complete clinical and transcriptomic data. While this substitution was necessary, we demonstrated comparable *ARSB/PDCD1* expression patterns between EONDGC and NDGC, supporting the feasibility of this approach. Nevertheless, potential biological heterogeneity cannot be ruled out (35, 36). Third, limited follow-up in the SYSU cohort (n = 171) may affect long-term metrics, yet 1-, 3-, and 5-year predictions remained robust. To address these limitations, future studies should incorporate broader datasets—such as Gene Expression Omnibus and institutional cohorts (e.g., SYSU)–and adopt prospective, multi-center designs to validate the APR and explore its immunotherapeutic potential. Our wet-lab validation was intentionally minimal—transient overexpression in two cell lines with qPCR confirmation only, scratch and Transwell migration assays, and no reciprocal knockdown/rescue or protein-level assays—so future work should incorporate loss-of-function/rescue experiments, protein validation, and pathway readouts to strengthen causal inference.

In summary, this study provides a comprehensive prognostic evaluation of EONDGC by integrating large-scale clinical data with molecular analysis. These findings contribute to the growing understanding of age-specific tumor biology and underscore the need for prospective, multicenter studies to further validate the APR index and explore its implications in personalized therapy and immuno-oncology.

## 5 Conclusion

This study pioneers the investigation of EONDGC by leveraging multiple datasets to develop a clinical prognostic model and identify *ARSB* and *PDCD1* as prognostic biomarkers. The *ARSB-PDCD1* ratio, a novel composite risk indicator, integrates these biomarkers to enhance risk stratification. These findings advance EONDGC molecular classification and support individualized prognosis.

## Data Availability

The original contributions presented in the study are included in the article/**Supplementary Material**. Further inquiries can be directed to the corresponding authors.
