# Supplementary material for: Prognostic modeling of early-onset nondistal gastric cancer identifies ARSB–PDCD1 ratio as an immune-related survival stratifier
Source: Front Immunol. 2025 Sep 29;16:1655106. doi: 10.3389/fimmu.2025.1655106 (PMC12515644; doi:10.3389/fimmu.2025.1655106)
Supplement: Supplementary file 1 [file Table1.docx]

**Table S1.**

| **Baseline characteristics of the SEER-training and SEER-validation sets** | | | |
| --- | --- | --- | --- |
| **Characteristics** | **SEER-Training Group** | **SEER-Validation Group** | P value |
|  | **N=376** | **N=159** |  |
| **Age of Diagnosis** | 44.0 [39.0;47.0] | 45.0 [40.5;47.0] | 0.341 |
| **Sex** |  |  | 0.334 |
| Female | 117 (31.1%) | 57 (35.8%) |  |
| Male | 259 (68.9%) | 102 (64.2%) |  |
| **Race** |  |  | 0.589 |
| White | 280 (74.5%) | 123 (77.4%) |  |
| Other | 57 (15.2%) | 24 (15.1%) |  |
| Black | 39 (10.4%) | 12 (7.55%) |  |
| **Pathological Pattern** |  |  | 0.468 |
| Adenocarcinoma | 278 (73.9%) | 123 (77.4%) |  |
| Signet ring | 98 (26.1%) | 36 (22.6%) |  |
| **Primary Site** |  |  | 0.44 |
| Cardia | 236 (62.8%) | 109 (68.6%) |  |
| Fundus | 30 (7.98%) | 11 (6.92%) |  |
| Body | 110 (29.3%) | 39 (24.5%) |  |
| **Pathological Grade** |  |  | 0.134 |
| Grade I | 15 (3.99%) | 4 (2.52%) |  |
| Grade II | 85 (22.6%) | 49 (30.8%) |  |
| Grade III | 267 (71.0%) | 100 (62.9%) |  |
| Grade IV | 9 (2.39%) | 6 (3.77%) |  |
| **AJCC Stage** |  |  | 0.566 |
| I | 68 (18.1%) | 26 (16.4%) |  |
| II | 101 (26.9%) | 39 (24.5%) |  |
| III | 141 (37.5%) | 70 (44.0%) |  |
| IV | 66 (17.6%) | 24 (15.1%) |  |
| **T Stage** |  |  | 0.72 |
| T1 | 52 (13.8%) | 18 (11.3%) |  |
| T2 | 111 (29.5%) | 47 (29.6%) |  |
| T3 | 156 (41.5%) | 73 (45.9%) |  |
| T4 | 57 (15.2%) | 21 (13.2%) |  |
| **N Stage** |  |  | 0.617 |
| N0 | 109 (29.0%) | 39 (24.5%) |  |
| N1 | 129 (34.3%) | 63 (39.6%) |  |
| N2 | 89 (23.7%) | 38 (23.9%) |  |
| N3 | 49 (13.0%) | 19 (11.9%) |  |
| **M Stage** |  |  | 0.485 |
| M0 | 331 (88.0%) | 144 (90.6%) |  |
| M1 | 45 (12.0%) | 15 (9.43%) |  |
| **Tumor Size (mm)** | 40.0 [25.0;60.0] | 41.0 [30.0;60.0] | 0.264 |
| **LNR** | 0.12 [0.00;0.42] | 0.13 [0.00;0.36] | 0.917 |
| **Type of Operation** |  |  | 0.826 |
| Partial Gastrectomy | 172 (45.7%) | 75 (47.2%) |  |
| Total Gastrectomy | 107 (28.5%) | 47 (29.6%) |  |
| Gastrectomy(NOS) | 97 (25.8%) | 37 (23.3%) |  |
| **Radiation** |  |  | 0.596 |
| No | 179 (47.6%) | 71 (44.7%) |  |
| Yes | 197 (52.4%) | 88 (55.3%) |  |
| **Chemotherapy** |  |  | 0.174 |
| No | 80 (21.3%) | 25 (15.7%) |  |
| Yes | 296 (78.7%) | 134 (84.3%) |  |
| **Marital Status** |  |  | 0.373 |
| Unmarried | 135 (35.9%) | 50 (31.4%) |  |
| Married | 241 (64.1%) | 109 (68.6%) |  |
| **Household Income** |  |  | 0.949 |
| ≤$69,999 | 218 (58.0%) | 91 (57.2%) |  |
| ＞$70,000 | 158 (42.0%) | 68 (42.8%) |  |
| **Survival Months** | 40.5 [17.0;90.0] | 41.0 [20.0;99.5] | 0.276 |
| **Vital Status** |  |  | 1 |
| Alive | 158 (42.0%) | 67 (42.1%) |  |
| Dead | 218 (58.0%) | 92 (57.9%) |  |
