# Supplementary material for: Prognostic modeling of early-onset nondistal gastric cancer identifies ARSB–PDCD1 ratio as an immune-related survival stratifier
Source: Front Immunol. 2025 Sep 29;16:1655106. doi: 10.3389/fimmu.2025.1655106 (PMC12515644; doi:10.3389/fimmu.2025.1655106)
Supplement: Supplementary file 2 [file Table2.docx]

| **Baseline information of the SEER-training and SYSU-validation sets** | | | |
| --- | --- | --- | --- |
| **Characteristics** | **SEER-Training Group** | **SYSU-Validation Group** | ***P* value** |
|  |  |  |  |
|  | **N=376** | **N=171** |  |
| **Age of Diagnosis** | 44.0 [39.0;47.0] | 42.0 [36.0;47.0] | 0.009 |
| **Race** |  |  | <0.001 |
| White | 280 (74.5%) | 1 (0.58%) |  |
| Other | 57 (15.2%) | 170 (99.4%) |  |
| Black | 39 (10.4%) | 0 (0.00%) |  |
| **Primary Site** |  |  | <0.001 |
| Cardia | 236 (62.8%) | 41 (24.0%) |  |
| Fundus of Stomach | 30 (7.98%) | 25 (14.6%) |  |
| Body of Stomach | 110 (29.3%) | 105 (61.4%) |  |
| **Pathological Grade** |  |  | <0.001 |
| Grade I | 15 (3.99%) | 4 (2.34%) |  |
| Grade II | 85 (22.6%) | 4 (2.34%) |  |
| Grade III | 267 (71.0%) | 53 (31.0%) |  |
| Grade IV | 9 (2.39%) | 110 (64.3%) |  |
| **T Stage** |  |  | <0.001 |
| T1 | 52 (13.8%) | 16 (9.36%) |  |
| T2 | 111 (29.5%) | 16 (9.36%) |  |
| T3 | 156 (41.5%) | 74 (43.3%) |  |
| T4 | 57 (15.2%) | 65 (38.0%) |  |
| **N Stage** |  |  | 0.002 |
| N0 | 109 (29.0%) | 59 (34.5%) |  |
| N1 | 129 (34.3%) | 42 (24.6%) |  |
| N2 | 89 (23.7%) | 30 (17.5%) |  |
| N3 | 49 (13.0%) | 40 (23.4%) |  |
| **M Stage** |  |  | <0.001 |
| M0 | 331 (88.0%) | 123 (71.9%) |  |
| M1 | 45 (12.0%) | 48 (28.1%) |  |
| **LNR** | 0.12 [0.00;0.42] | 0.10 [0.00;0.35] | 0.644 |
| **Chemotherapy** |  |  | <0.001 |
| No | 80 (21.3%) | 93 (54.4%) |  |
| Yes | 296 (78.7%) | 78 (45.6%) |  |
| **Survival Months** | 40.5 [17.0;90.0] | 25.1 [10.4;56.0] | <0.001 |
| **Vital Status** |  |  | 0.282 |
| Alive | 158 (42.0%) | 81 (47.4%) |  |
| Dead | 218 (58.0%) | 90 (52.6%) |  |

**Table S2.**
