# Supplementary material for: Prognostic modeling of early-onset nondistal gastric cancer identifies ARSB–PDCD1 ratio as an immune-related survival stratifier
Source: Front Immunol. 2025 Sep 29;16:1655106. doi: 10.3389/fimmu.2025.1655106 (PMC12515644; doi:10.3389/fimmu.2025.1655106)
Supplement: Supplementary file 3 [file Table3.docx]

**Table S3.**

| **The scores of clinical variables in EONDGC model and the corresponding total risk points and estimated OS probabilities** | | | | | | | | |
| --- | --- | --- | --- | --- | --- | --- | --- | --- |
|  |  |  |  |  | **Total Points** | | |  |
| **Characteristics** | **Points** | **Characteristics** | **Points** |  | **1-Year** | **3-Year** | **5-Year** | **Overall Survival** |
| **Race** |  | **M Stage** |  |  | 201 | 111 | 89 | 0.90 |
| White | 32 | M0 | 0 |  |  |  |  |  |
| Other | 0 | M1 | 55 |  | 226 | 136 | 114 | 0.85 |
| Black | 25 | **Chemotherapy** |  |  |  |  |  |  |
| **Pathological Grade** |  | No | 38 |  | 244 | 154 | 133 | 0.80 |
| Grade I | 0 | Yes | 0 |  |  |  |  |  |
| Grade II | 33 | **LNR** |  |  | 272 | 182 | 160 | 0.70 |
| Grade III | 46 | [0.0,0.1) | [0,8) |  |  |  |  |  |
| Grade IV | 80 | [0.1,0.2) | [8,17) |  | 292 | 203 | 181 | 0.60 |
| **T Stage** |  | [0.2,0.3) | [17,25) |  |  |  |  |  |
| T1 | 0 | [0.3,0.4) | [25,34) |  | 310 | 220 | 199 | 0.50 |
| T2 | 62 | [0.4,0.5) | [34,42) |  |  |  |  |  |
| T3 | 77 | [0.5,0.6) | [42,51) |  | 326 | 236 | 215 | 0.40 |
| T4 | 100 | [0.6.0.7) | [51,59) |  |  |  |  |  |
| **N Stage** |  | [0.7,0.8) | [59,68) |  | 342 | 252 | 231 | 0.30 |
| N0 | 0 | [0.8,0.9) | [68,76) |  |  |  |  |  |
| N1 | 44 | [0.9,1.0] | [76,85] |  | 359 | 269 | 248 | 0.20 |
| N2 | 52 |  |  |  |  |  |  |  |
| N3 | 55 |  |  |  | 380 | 290 | 268 | 0.10 |
