# Supplementary material for: Prognostic modeling of early-onset nondistal gastric cancer identifies ARSB–PDCD1 ratio as an immune-related survival stratifier
Source: Front Immunol. 2025 Sep 29;16:1655106. doi: 10.3389/fimmu.2025.1655106 (PMC12515644; doi:10.3389/fimmu.2025.1655106)
Supplement: Supplementary file 4 [file Table4.docx]

**Table S4.**

| **Prognostic value of 73 upregulated genes in TCGA–NDGC: univariate and multivariate Cox regression analyses** | | | | | | | |
| --- | --- | --- | --- | --- | --- | --- | --- |
| **Gene** | **Univariate Analysis** | | |  | **Multivariate Analysis** | | |
|  | **HR** | **95% CI** | P value |  | **HR** | **95% CI** | P value |
| *SERPING1* | 1.09 | 0.67-1.76 | 0.727 |  |  |  |  |
| *APOE* | 1.36 | 0.84-2.21 | 0.208 |  |  |  |  |
| *TNC* | 1.7 | 1.04-2.79 | 0.034 |  |  |  |  |
| *GPNMB* | 1.57 | 0.96-2.57 | 0.073 |  |  |  |  |
| *CXCL9* | 0.65 | 0.4-1.06 | 0.087 |  | 0.65 | 0.36-1.15 | 0.137 |
| *PPP1R18* | 1.38 | 0.85-2.25 | 0.195 |  |  |  |  |
| *ITPKB* | 1.17 | 0.72-1.89 | 0.521 |  |  |  |  |
| *PTPN14* | 1.55 | 0.94-2.54 | 0.083 |  |  |  |  |
| *WWC3* | 1.88 | 1.14-3.12 | 0.014 |  |  |  |  |
| *FCGR3A* | 1.32 | 0.82-2.14 | 0.257 |  |  |  |  |
| *JCAD* | 2.02 | 1.21-3.37 | 0.007 |  |  |  |  |
| *TSPAN9* | 1.19 | 0.73-1.94 | 0.481 |  |  |  |  |
| *COMP* | 1.44 | 0.88-2.35 | 0.144 |  |  |  |  |
| *PBX3* | 1.6 | 0.98-2.61 | 0.061 |  |  |  |  |
| *CACNA1C* | 1.35 | 0.83-2.19 | 0.227 |  |  |  |  |
| *AP1S2* | 1.43 | 0.87-2.33 | 0.155 |  |  |  |  |
| *FPR3* | 1.04 | 0.64-1.69 | 0.876 |  |  |  |  |
| *F13A1* | 1.35 | 0.82-2.21 | 0.236 |  |  |  |  |
| *CD177* | 1.51 | 0.92-2.48 | 0.102 |  |  |  |  |
| *NAB2* | 1.4 | 0.86-2.28 | 0.178 |  |  |  |  |
| *ARSB* | 2.23 | 1.33-3.72 | 0.002 |  | 2.4 | 1.38-4.17 | 0.002 |
| *MRC1* | 0.9 | 0.56-1.45 | 0.663 |  |  |  |  |
| *IGSF9B* | 0.93 | 0.58-1.51 | 0.782 |  |  |  |  |
| *RAB23* | 1.48 | 0.9-2.41 | 0.120 |  |  |  |  |
| *IDS* | 0.99 | 0.61-1.61 | 0.980 |  |  |  |  |
| *CMKLR1* | 0.98 | 0.61-1.59 | 0.948 |  |  |  |  |
| *FCMR* | 1.04 | 0.65-1.69 | 0.862 |  |  |  |  |
| *ETV1* | 1.33 | 0.82-2.17 | 0.246 |  |  |  |  |
| *IL17RD* | 0.82 | 0.5-1.32 | 0.412 |  |  |  |  |
| *ATP10A* | 1.09 | 0.67-1.76 | 0.728 |  |  |  |  |
| *NKG7* | 0.82 | 0.51-1.34 | 0.432 |  |  |  |  |
| *HHEX* | 0.87 | 0.54-1.42 | 0.579 |  |  |  |  |
| *AKAP6* | 0.92 | 0.57-1.48 | 0.722 |  |  |  |  |
| *CCL19* | 1.55 | 0.95-2.52 | 0.080 |  | 1.58 | 0.92-2.73 | 0.1 |
| *SLC1A3* | 1.08 | 0.66-1.74 | 0.769 |  |  |  |  |
| *SMYD1* | 1.23 | 0.76-1.99 | 0.399 |  |  |  |  |
| *GNLY* | 0.66 | 0.4-1.09 | 0.102 |  |  |  |  |
| *S1PR2* | 1 | 0.62-1.61 | 0.986 |  |  |  |  |
| *CLEC2B* | 0.71 | 0.44-1.16 | 0.176 |  |  |  |  |
| *CCR7* | 0.97 | 0.6-1.57 | 0.905 |  |  |  |  |
| *SUCNR1* | 0.74 | 0.46-1.21 | 0.232 |  |  |  |  |
| *SHISA3* | 0.97 | 0.6-1.58 | 0.911 |  |  |  |  |
| *ITIH2* | 1.47 | 0.91-2.38 | 0.117 |  |  |  |  |
| *SCRG1* | 1.15 | 0.71-1.86 | 0.581 |  |  |  |  |
| *LY86* | 1.29 | 0.79-2.1 | 0.309 |  |  |  |  |
| *HVCN1* | 1.04 | 0.64-1.69 | 0.867 |  |  |  |  |
| *CACNA1E* | 0.94 | 0.58-1.52 | 0.807 |  |  |  |  |
| *P2RX7* | 1.05 | 0.65-1.7 | 0.847 |  |  |  |  |
| *CD226* | 0.87 | 0.54-1.4 | 0.560 |  |  |  |  |
| *TLR8* | 0.94 | 0.58-1.52 | 0.795 |  |  |  |  |
| *CLEC10A* | 0.94 | 0.57-1.53 | 0.792 |  |  |  |  |
| *EOMES* | 0.83 | 0.51-1.35 | 0.448 |  |  |  |  |
| *CTLA4* | 0.67 | 0.41-1.1 | 0.114 |  |  |  |  |
| *IGF1* | 1.19 | 0.73-1.94 | 0.479 |  |  |  |  |
| *DKK2* | 1.19 | 0.74-1.93 | 0.478 |  |  |  |  |
| *PDCD1* | 0.63 | 0.38-1.03 | 0.066 |  | 0.5 | 0.27-0.91 | 0.024 |
| *ST8SIA1* | 1.38 | 0.85-2.23 | 0.197 |  |  |  |  |
| *FMO1* | 1.66 | 1.01-2.71 | 0.044 |  |  |  |  |
| *ZNF683* | 0.93 | 0.57-1.51 | 0.762 |  |  |  |  |
| *TRAT1* | 0.88 | 0.54-1.44 | 0.611 |  |  |  |  |
| *FGF9* | 1.52 | 0.94-2.48 | 0.090 |  | 1.44 | 0.88-2.37 | 0.149 |
| *CLEC12A* | 1.1 | 0.68-1.79 | 0.696 |  |  |  |  |
| *GIMAP5* | 0.84 | 0.52-1.36 | 0.479 |  |  |  |  |
| *IGFN1* | 1.19 | 0.74-1.93 | 0.474 |  |  |  |  |
| *GRPR* | 1.16 | 0.72-1.88 | 0.548 |  |  |  |  |
| *CCL23* | 0.97 | 0.6-1.58 | 0.918 |  |  |  |  |
| *CASQ1* | 1.36 | 0.83-2.21 | 0.218 |  |  |  |  |
| *GRAP* | 0.87 | 0.54-1.41 | 0.579 |  |  |  |  |
| *LILRA4* | 1.45 | 0.89-2.35 | 0.135 |  |  |  |  |
| *ALK* | 0.91 | 0.56-1.46 | 0.686 |  |  |  |  |
| *TMEFF2* | 1.1 | 0.68-1.78 | 0.698 |  |  |  |  |
| *C4orf50* | 0.89 | 0.55-1.44 | 0.636 |  |  |  |  |
| *KIR3DL1* | 0.99 | 0.61-1.6 | 0.951 |  |  |  |  |
