# Supplementary figures and images for: Prognostic modeling of early-onset nondistal gastric cancer identifies ARSB–PDCD1 ratio as an immune-related survival stratifier
Source: Front Immunol. 2025 Sep 29;16:1655106. doi: 10.3389/fimmu.2025.1655106 (PMC12515644; doi:10.3389/fimmu.2025.1655106)

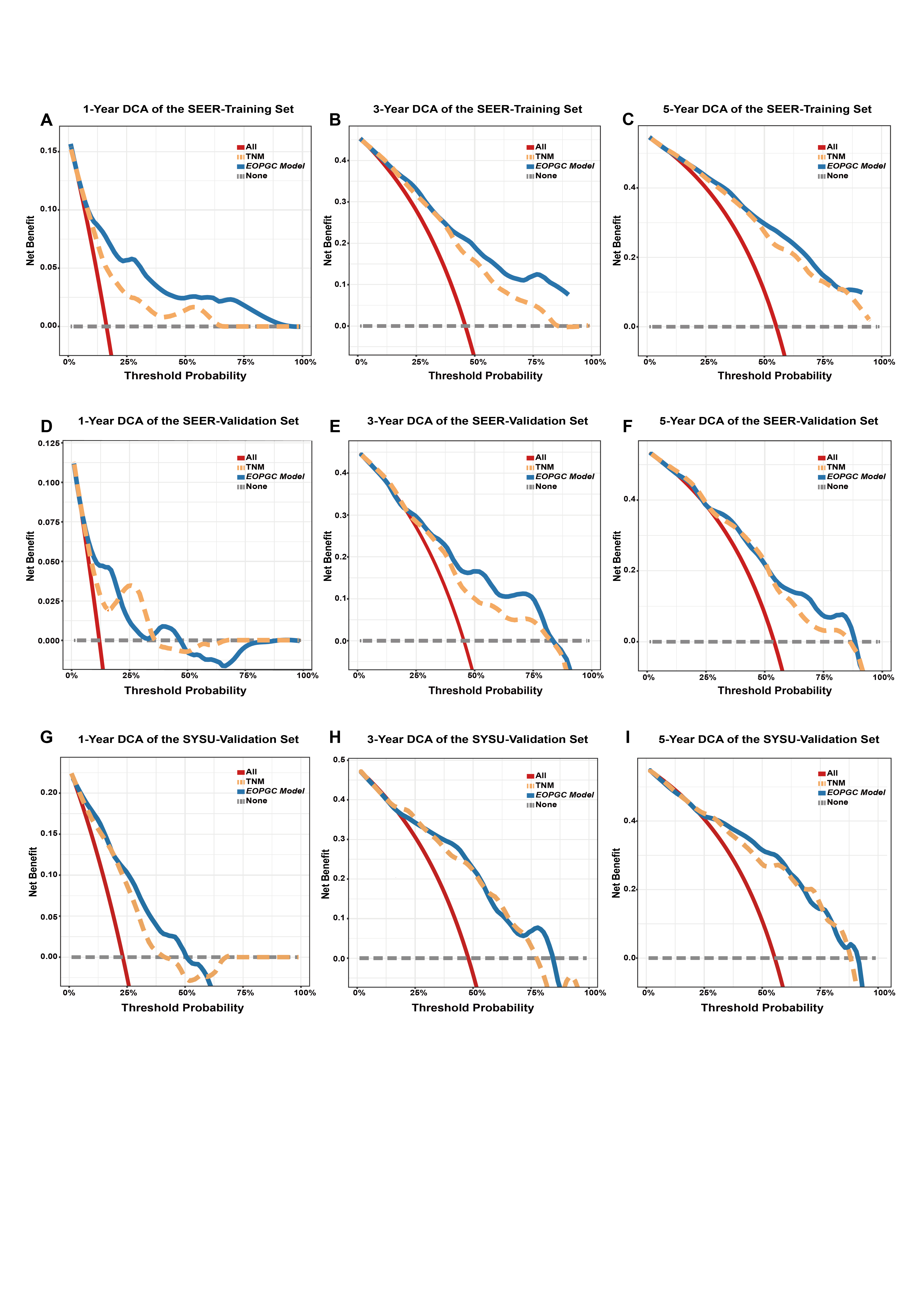

Supplement: Supplementary file 5 [file Image1.jpeg]

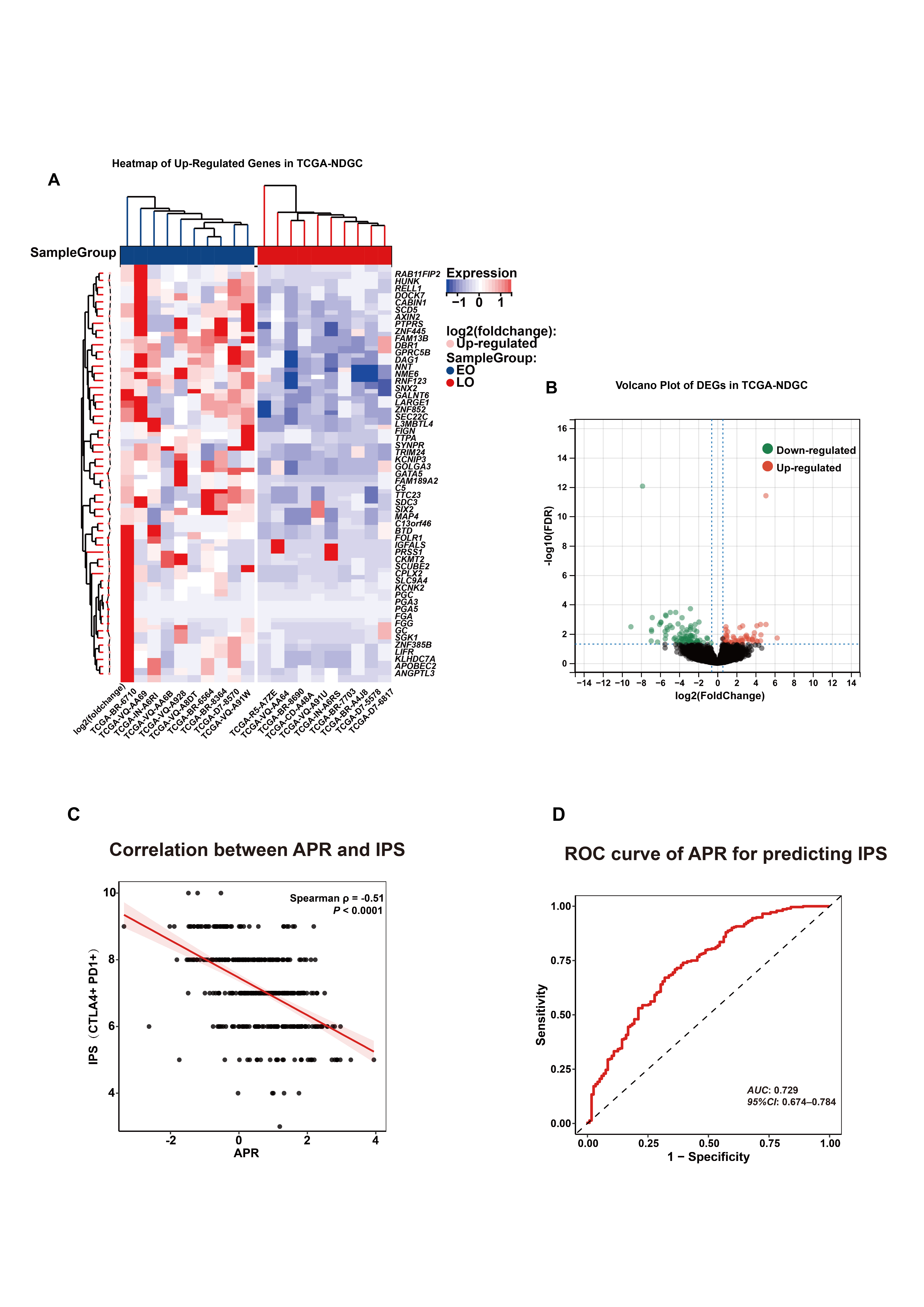

Supplement: Supplementary file 6 [file Image2.jpeg]
